# Recombinant neutralizing secretory IgA antibodies for preventing mucosal acquisition and transmission of SARS-CoV-2

**DOI:** 10.1016/j.ymthe.2024.01.025

**Published:** 2024-01-24

**Authors:** Kathrin Göritzer, Elisabetta Groppelli, Clemens Grünwald-Gruber, Rudolf Figl, Fengfeng Ni, Huimin Hu, Yuncheng Li, Yalan Liu, Qinxue Hu, Rama Devudu Puligedda, Jae-Wan Jung, Richard Strasser, Scott Dessain, Julian K.-C. Ma

**Affiliations:** 1 Hotung Molecular Immunology Unit, St. George’s University of London, London SW17 0RE, UK; 2 Institute for Infection and Immunity, St. George’s University of London, London SW17 0RE, UK; 3 Core Facility Mass Spectrometry, University of Natural Resources and Life Sciences, 1190 Vienna, Austria; 4 State Key Laboratory of Virology, Wuhan Institute of Virology, Center for Biosafety Mega-Science, Chinese Academy of Sciences, Wuhan 430071, China; 5 Lankenau Institute for Medical Research, Wynnewood, PA 19096, USA; 6 Department of Biochemistry and Metabolism, John Innes Centre, Norwich Research Park, Norwich NR4 7UH, UK; 7 Department of Applied Genetics and Cell Biology, University of Natural Resources and Life Sciences, 1190 Vienna, Austria

**Keywords:** SARS-CoV-2, monoclonal antibodies, virus neutralization, mucosal antibodies, secretory IgA, passive immunization, topical delivery, molecular pharming, *Nicotiana benthamiana*

## Abstract

Passive delivery of antibodies to mucosal sites may be a valuable adjunct to COVID-19 vaccination to prevent infection, treat viral carriage, or block transmission. Neutralizing monoclonal IgG antibodies are already approved for systemic delivery, and several clinical trials have been reported for delivery to mucosal sites where SARS-CoV-2 resides and replicates in early infection. However, secretory IgA may be preferred because the polymeric complex is adapted for the harsh, unstable external mucosal environment. Here, we investigated the feasibility of producing neutralizing monoclonal IgA antibodies against SARS-CoV-2. We engineered two class-switched mAbs that express well as monomeric and secretory IgA (SIgA) variants with high antigen-binding affinities and increased stability in mucosal secretions compared to their IgG counterparts. SIgAs had stronger virus neutralization activities than IgG mAbs and were protective against SARS-CoV-2 infection in an *in vivo* murine model. Furthermore, SIgA1 can be aerosolized for topical delivery using a mesh nebulizer. Our findings provide a persuasive case for developing recombinant SIgAs for mucosal application as a new tool in the fight against COVID-19.

## Graphical abstract

**Figure:**
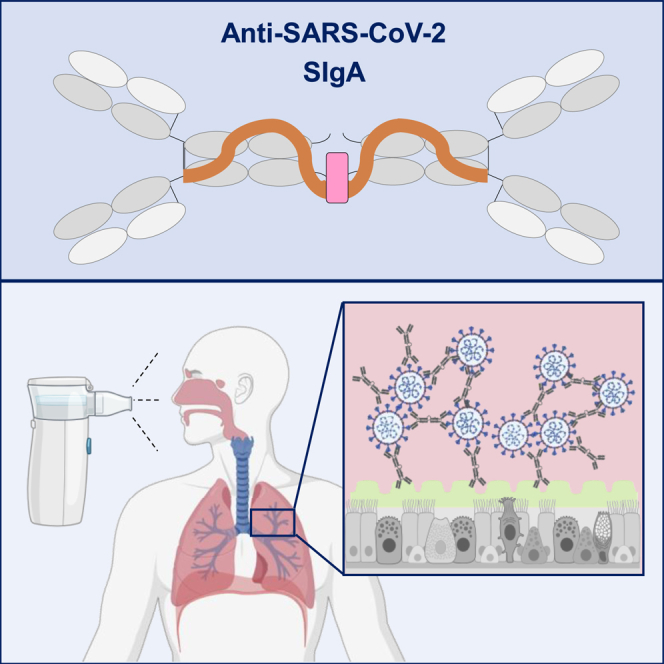

## Teaser

Göritzer and colleagues investigate secretory IgA (SIgA) for mucosal delivery, complementing COVID-19 vaccination. SIgA demonstrates superior virus neutralization over IgG, proving protective in murine models. Aerosolizable for topical delivery via a mesh nebulizer, SIgAs have emerged as a promising tool to combat SARS-CoV-2.

## Introduction

Coronavirus disease 2019 (COVID-19) is a mucosal infection caused by severe acute respiratory syndrome-coronavirus-2 (SARS-CoV-2). The virus replicates in the respiratory tract and is transmitted through respiratory droplets produced when an infected person coughs, sneezes, or talks. The most prominent symptoms of COVID-19 affect the respiratory system (continuous cough, shortness of breath), but in some cases sensory tissues in the upper respiratory tract are involved, causing anosmia and loss of taste.^1^ In addition, gastrointestinal (GI) symptoms (nausea, vomiting, and diarrhea) are reported in 6% of adults and up to 20% of children.^2^ Virus can be detected at all of these sites as well as in urine.^3^

Infection with SARS-CoV-2 elicits systemic and mucosal immune responses.^4^ Although attention has been focused on serum antibody responses that are dominated by immunoglobulin G (IgG), at mucosal sites such as the respiratory, GI, and genitourinary tracts, IgA in the external secretions that bathe mucosal surfaces is the predominant antibody class.^5^ Mucosal IgA in SARS-CoV-2 can be neutralizing and long-lasting.^4^

Various comorbidities have been associated with diminished immune responses to SARS-CoV-2, including immunosuppressive drugs to prevent transplant failure and diabetes.^6^ Seroconversion following COVID-19 vaccination can also be compromised in these and similar patients.^7^^,^^8^ In such circumstances, passive delivery of antibodies may be a valuable adjunct to COVID-19 vaccination, in which neutralizing antibodies could be delivered directly to mucosal sites either to prevent infection, treat viral carriage, or block transmission. Furthermore, the topical delivery of antibodies could be useful to prevent the carriage of virus in asymptomatic individuals.

Neutralizing monoclonal IgG antibodies are already approved for systemic use in early SARS-CoV-2 treatment, and several clinical trials have been reported for the topical delivery of IgG to the lungs to treat viral infections, including SARS-CoV-2.^9^^,^^10^^,^^11^ These have not always met with success and, notably, Boehringer Ingelheim stopped a Phase II clinical trial of its inhaled IgG antibody due to lack of efficacy.

For mucosal sites, IgA in the form of secretory IgA (SIgA), may be preferred because the polymeric complex is adapted for harsh, unstable external mucosal environments, which are nonsterile and rich in endogenous and exogenous proteases.^12^^,^^13^ SIgA exists as a dimer consisting of two IgAs joined by a joining chain (JC), and is further complexed with a secretory component (SC) to form SIgA. This SC is hydrophilic and a highly glycosylated negatively charged molecule that protects SIgA from degradation in luminal secretions. In contrast to IgG, SIgA is generally considered to be a noninflammatory neutralizing antibody by promoting the clearance of pathogens from mucosal surfaces by blocking access to epithelial receptors, trapping them in mucus, and facilitating their removal by peristaltic mucociliary activities.^14^ The potency and efficacy of recombinant monoclonal IgA mAbs (monoclonal antibodies) for protection against different disease through topical delivery has been well established for mothers’ milk and vaginal, nasal, and GI secretions.^15^^,^^16^ The potential of the IgA isotype mAbs for direct administration has also been explored.^17^^,^^18^^,^^19^^,^^20^^,^^21^^,^^22^

Monoclonal SIgA antibodies are technically challenging to produce, however. The first recombinant approach to expressing secretory antibodies was in genetically modified plants.^23^ Other approaches have been described, but they still seem impractical or unaffordable for commercial development.^24^^,^^25^^,^^26^ Some improvements to SIgA expression have been reported in plants, which still seems the most promising approach.^27^^,^^28^^,^^29^^,^^30^

In this study, we investigated the feasibility of producing neutralizing monoclonal IgA antibodies against SARS-CoV-2. Starting with different published, well-characterized, and strongly neutralizing IgG class mAbs targeting different epitopes of the receptor-binding domain (RBD) and N-terminal domain (NTD) domains of SARS-CoV-2, we expressed monomeric and secretory forms of IgA and compared these for their functionality and stability.^31^^,^^32^^,^^33^ Finally, we assessed the potential use of SIgA to prevent SARS-CoV-2 infection in an *in vivo* model.

## Results

### Recombinant production of anti-SARS-CoV-2 mucosal antibodies in plants

**Figure 1:**
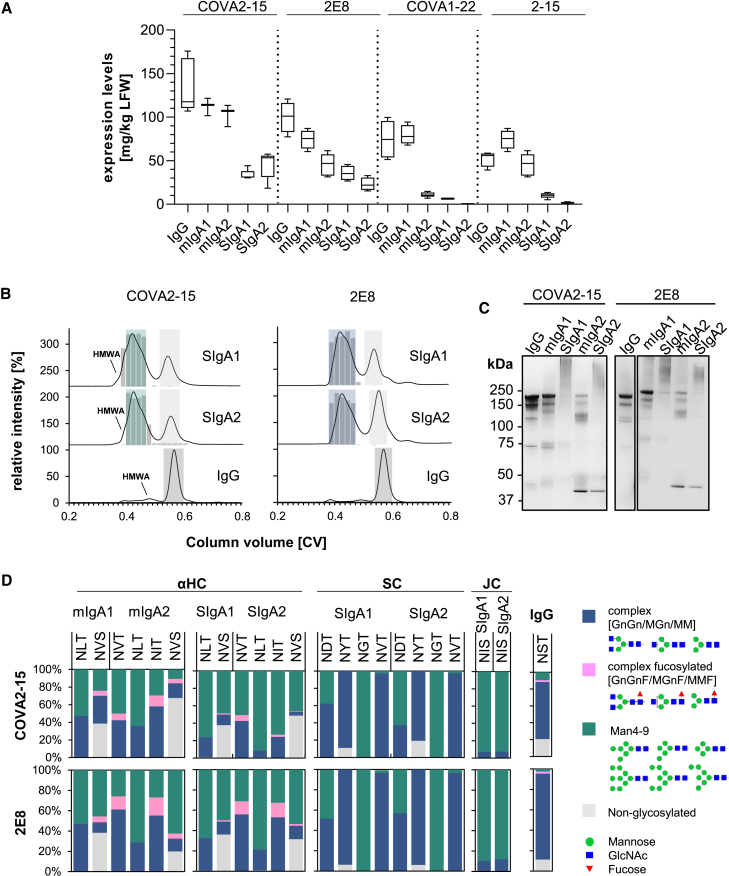
Expression, assembly, and glycosylation of monoclonal IgG and different IgA antibodies from *N. benthamiana* plants (A) IgG and monomeric and secretory IgA1 and IgA2 versions of 4 different mAbs recognizing the SARS-CoV-2 Spike proteins were transiently expressed in plants. Expression levels were quantified by sandwich ELISA in crude leaf extracts. The detection of monomeric IgA and IgG variants was with either HRP-labeled anti-kappa (COVA2-15) or anti-lambda light-chain (2E8, COVA1-22, 2–15) antibodies. SIgA antibodies were detected using anti-SC antibodies for all SIgA variants. Quantification data represent the mean of 2 technical repeats of 3 independent infiltrations of 3 plants each ± SD. (B) Normalized size-exclusion chromatograms of affinity-purified IgG, secretory IgA1, and secretory IgA2 of the COVA2-15 and 2E8 variants from infiltrated *N. benthamiana* ΔXT/FT leaves. Values were normalized based on the highest signal of each chromatogram. The ratio of functional secretory IgA to total IgA in each chromatography fraction was determined by antigen sandwich ELISA, and the relative amount of functional SIgA in each fraction is indicated by gray bars. Green, blue, and gray boxes indicated pooled fractions. (C) SDS-PAGE under nonreducing conditions of affinity and size-exclusion purified plant-produced IgG and monomeric and secretory IgA1/IgA2 of COVA2-15 and 2E8 visualized by Coomassie brilliant blue staining. (D) Site-specific *N*-glycosylation of purified mAbs. Bars represent the relative abundance (%) of glycoforms present at each glycosite of the heavy chains (HC; IgA1: sequon NLT and NVS, IgA2: NVT, NLT, NIT, and NVS, IgG1: NST), the SC (NTD, NYT, NGT, and NVT) and the JC (NIS). N-Glycans are abbreviated according to the ProGlycAn system (www.proglycan.com). The symbols for the monosaccharides are drawn according to the nomenclature from the Consortium for Functional Glycomics.

We generated monomeric and secretory IgA1 and IgA2 versions of four individual SARS-CoV-2 neutralizing IgG mAbs targeting different epitopes on the Spike protein. The variable regions of the well-characterized, neutralizing SARS-CoV-2-specific IgG antibodies COVA2-15, COVA1-22, 2-15, and 2E8 were cloned onto IgA1 and IgA2 constant domains for transient expression in glycoengineered *Nicotiana benthamiana* ΔXT/FT plants that are almost completely deficient in β1,2-xylosylation and core α1,3-fucosylation.^31^^,^^33^^,^^34^ Light- and heavy-chain pairs were coexpressed in the presence and absence of the JC and SC to obtain either monomeric or secretory IgA. Immunoblot analysis and ELISA showed the highest accumulation of recombinant protein after 5 to 6 days postinfiltration. The expression levels of all monomeric IgA1 (mIgA1) and IgA2 (mIgA2) variants were high and approached those of their IgG counterparts (∼100 mg/kg leaf fresh weight [LFW] (Figure 1A; Table S1), and all of them were functional in terms of binding to the SARS-CoV-2 Spike protein (Figure S1). However, assembly into multimeric secretory IgA when the JC and SC were coexpressed resulted in a significant yield loss. COVA2-15 SIgA was expressed at 33–45 mg/kg LFW and 2E8 SIgA at 23–35 mg/kg LFW, but the yields for COVA1-22 and 2–15 were most affected (1–10 mg/kg LFW) (Figure 1A). COVA2-15 and 2E8 mAbs were therefore selected for further analysis and characterization.

After affinity purification, all of the IgG and IgA isotypes of COVA2-15 and 2E8 were subjected to size-exclusion chromatography (SEC). Both COVA2-15 and 2E8 IgG variants display single monodispersed peaks at the expected retention time for proteins with a mass of ∼150 kDa (Figure 1B, dark gray shaded area). COVA2-15 and 2E8 monomeric IgA variants also displayed a major peak corresponding to the monomeric structural unit, with additional minor peaks at lower retention times representing high-molecular-weight aggregates (HMWA) (Figure S2). Co-infiltration of IgA with the JC and SC resulted in a major peak with minor shoulders at earlier retention times (Figure 1B, green/blue shaded area) as well as a second peak representing nonassembled monomeric IgA (Figure 1B, light shaded area). Each of the eluted fractions was analyzed by ELISA to determine the ratio of fully functional and assembled secretory IgA (Figure 1B, gray bars). Recombinant IgAs were captured with RBD and detected with anti-SC antibody and compared to total IgA by using an anti-IgA heavy-chain antibody for capture and an anti-kappa or lambda light-chain antibody for detection. Thus, it was shown that the major peak and its shoulder at higher retention time (green/blue shaded area) of all of the variants contains fully assembled and functional SIgA, whereas the peak shoulder observed for COVA2-15 SIgA1 and SIgA2 at an earlier retention time likely contains nonfunctional HMWA. In general, the formation of multimeric COVA2-15 and 2E8 IgA variants was very efficient compared to COVA1-22 and 2-15 (Figure S3) and previous reports of other multimeric IgA variants produced in plants, whereas COVA2-15 SIgA1 and SIgA2 displayed better assembly than their 2E8 counterparts.^27^^,^^29^^,^^30^ The overall purification yields of fully assembled and functional SIgA after affinity chromatography and SEC were between 10 and 42 mg/kg LFW (Table S2).

To elucidate whether such yields could be maintained at a larger scale, COVA2-15 SIgA1 and 2E8 SIgA1 were manufactured using a 500-g pilot-scale production process by an independent contract manufacturer (Leaf Expression Systems, Norwich, UK) (Figure S4). The expression levels of SIgA at 5 days posttransfection were slightly improved to 50–75 mg/kg LFW, whereas the ratio of SIgA to monomeric IgA was similar to our lab-scale experiments. The purification yields after SEC of fully assembled and functional SIgA were 50 mg/kg LFW for COVA2-15 SIgA1 and 25.5 mg/kg LFW for 2E8 SIgA1.

SEC fractions containing either the secretory or monomeric IgA species were pooled and were further analyzed using SDS-PAGE (Figure 1C). Under nonreducing conditions COVA2-15 and 2E8 IgG display 2 predominant bands at 150 kDa, representing fully glycosylated and underglycosylated mAbs commonly observed in plant-produced IgG. Both COVA2-15 and 2E8 mIgA1 and mIgA2 show a predominant band at a molecular mass of ∼160 kDa representing the fully assembled monomer. Monomeric IgA2 variants displayed additional bands at ∼100 and 45 kDa, which likely represent heavy- and light-chain dimers because the IgA2m(1) isotype used here does not have disulfide bridges linking the heavy and light chains, which are only associated through noncovalent intermolecular interactions.^35^ Secretory IgA1 and IgA2 variants display a predominant broad band at the expected size of 360–400 kDa. The additional bands observed for monomeric IgA2 were not observed to the same extent for secretory IgA2.

### Glycosylation of plant-produced mucosal antibodies

SIgA is a heavily glycosylated protein with two predicted *N*-glycosylation sites in the IgA1 heavy chain, four in the IgA2m(1) heavy chain, one in the joining chain and six in the SC. In addition, IgA1 has nine potential *O*-glycosylation sites in the proline-rich hinge region. To assess the glycosylation status of plant-produced IgG, monomeric IgA, and secretory IgA isotypes, the purified antibody variants were digested with trypsin and subjected to liquid chromatography (LC)-electrospray ionization-mass spectrometry (MS) for analysis of site-specific *N*-glycosylation and the presence of modifications within the IgA1 hinge region (Figures 1D and S5; Tables S3–S5). The single *N*-glycosylation site in the IgG heavy chain of COVA2-15 and 2E8 was ∼90% occupied and displayed a very homogeneous glycosylation profile with the fully processed biantennary complex-type GlcNAc2Man3GlcNAc2 (GnGn) as a major glycoform and lesser amounts of GlcNAc1Man3GlcNAc2 (MGn/GnM).

All of the *N*-glycosylation sites in monomeric and secretory IgA1 and IgA2 heavy chains were fully occupied except for the C-terminal *N*-site present in the tailpiece of IgA (sequon NVS), which was only 30%–70% glycosylated, as previously reported for plant-produced IgA.^35^ In addition to biantennary complex-type structures (GnGn, MGn), the IgA heavy chains contained high amounts of oligomannosidic (Man5–9) and paucimannosidic (MM) structures, as well as small amounts of complex *N*-glycans carrying the plant-specific core α1,3-fucose resulting from the incomplete silencing of α1,3-fucosyltransferase in the *N. benthamiana* ΔXT/FT line. Furthermore, some site-specific processing can be observed for the *N*-site in the CH2 domain (sequon NLT) of IgA1 and IgA2, which completely lacks α1,3-fucose and displays high amounts of oligomannosidic structures. The incomplete processing suggests inaccessibility of the *N*-glycans for the respective glycosyltransferases, which is even more pronounced when the SC is incorporated.

We were able to detect the single glycopeptide corresponding to the JC of the secretory IgA variants (Figure 1D; Table S4). The single *N*-glycan site in the JC of all of the variants was almost fully occupied and consisted of oligomannosidic structures, which differs from the very heterogeneously glycosylated JC of mammalian-produced SIgA containing complex-type glycans with high levels of branching and incomplete sialylation.^30^ The presence of oligomannosidic *N*-glycans suggests incomplete processing of the JC *N*-glycans in the Golgi of plants.

Furthermore, we were able to identify four individual tryptic glycopeptides of the SC, which were all fully occupied and displayed site-specific glycan processing (Figure 1D; Table S4). There was little difference between SC glycosylation of SIgA1 and SIgA2, or between COVA2-15 and 2E8 SIgAs. *N*-site at sequon NGT exclusively contained oligomannosidic *N*-glycans indicating reduced accessibility for processing at this site. Sites NTD, NYT, and NVT consisted mostly of complex-type biantennary and paucimannosidic structures (MM > GnGn, MGn), which are likely generated in a post-Golgi compartment by β-hexosaminidases and completely lacked plant-specific α1,3-fucose.^36^ On the hinge region of the plant-produced monomeric and secretory IgA1 we detected the conversion of up to six proline residues to hydroxyproline and the addition of variable amounts of arabinoses in 30%–50% of the converted hinge regions (Figure S5; Table S5).

### Stability and interaction of anti-SARS-CoV-2 mAbs in human saliva

**Figure 2:**
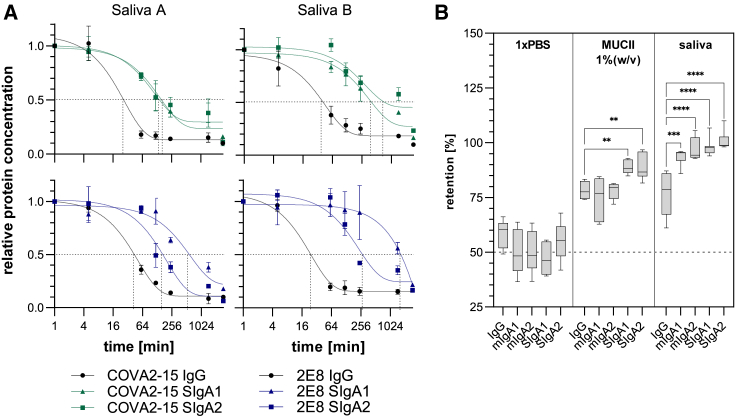
Stability and interaction of anti-SARS-CoV-2 IgG and IgA with mucus (A) Saliva from 2 donors (Saliva A and Saliva B) was mixed with COVA2-15 and 2E8 IgG and SIgA mAb variants and incubated at 37°C for the indicated time. Samples were analyzed for binding to RBD and assembly through detection with Fc-specific and SC-specific antibodies. The mean ± SD of duplicates is shown. Gray dotted lines indicate half-lives of COVA2-15 and 2E8 variants calculated using a 1-phase decay nonlinear regression model. (B) COVA2-15 IgG and IgA mAbs were mixed with PBS, MUCII or human saliva and dialyzed against PBS using a 0.05-μm filter in a fast equilibrium microdialysis setup. mAb concentrations pre- and postdialysis were quantified via antigen sandwich ELISA. Data represent the mean of at least 4 repeats ± SEM. One-way ANOVA was performed to compare IgA groups with the IgG group; ∗∗p < 0.01; ∗∗∗p < 0.001; ∗∗∗∗p < 0.0001.

Due to particular structural features SIgA is expected to be better suited than IgG to survive and function on mucosal surfaces.^15^^,^^37^ To evaluate the stability of plant-produced anti-SARS-CoV-2 IgG and secretory IgA variants in human secretions, an *in vitro* experiment with COVA2-15 and 2E8 IgG, SIgA1, and SIgA2 was performed using saliva from two donors (Figure 2A). Each mAb variant was incubated with clarified saliva, incubated at 37°C, and sampled at the times indicated. Time point samples were analyzed for structural integrity and retained antigen-binding capacity by sandwich ELISA capturing using RBD and detection with horseradish peroxidase (HRP)-conjugated anti-IgG-Fc or anti-SC. Although the rates of degradation for both IgG and IgA variants based on COVA2-15 and 2E8 varied between experiments when different saliva samples were used, intact IgG was lost at a consistently faster rate than secretory IgA variants over the experimental time course. The half-lives of the SARS-CoV-2 IgG mAbs were calculated using a one-phase decay nonlinear regression model. The half-lives of COVA2-15 and 2E8 IgG variants were between 15 and 30 min. In contrast, the half-lives of COVA2-15 SIgA1 and SIgA2 and 2E8 SIgA2 were increased 5- to 10-fold to up to 200 min. The half-life for 2E8 SIgA1 was difficult to determine because values did not decline to a plateau in the tested time frame.

To test the interaction of different plant-produced Ig formats with mucus, microdialysis experiments were performed (Figure 2B). Recombinant mAbs were incubated with either mucin type II (MUCII) from porcine or human saliva supernatant from a single donor, which was negative for mucosal SARS-CoV-2-specific IgG/IgA and dialyzed against PBS for 16 h using a membrane with a cutoff of 0.05 μm. Pores of this size allow Igs to exit the sample chamber, whereas macromolecular complexes within the mucin network are retained. The distribution of Igs between sample and dialysis chamber pre- and postdialysis were determined by ELISA. All Ig formats showed some degree of association with MUCII and saliva. However, compared to IgG that has a median retention of 75% in both MUCII and saliva, mIgAs showed similar retention in MUCII and high retention of ∼95% in saliva. The highest association with both MUCII and saliva was observed for secretory IgA variants with up to 90% and 99% retention, respectively.

### Binding characteristics of different antibody formats to SARS-CoV-2 Spike protein

**Figure 3:**
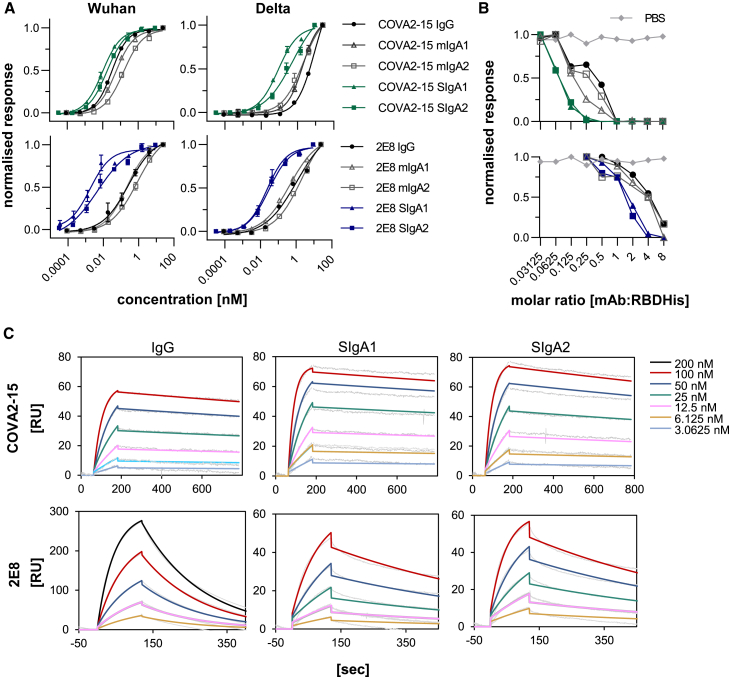
Interaction of CoVA2-15 and 2E8 IgG and IgA antibodies with the SARS-CoV-2 RBD (Α) Determination of EC_50_ values of IgA and IgG anti-SARS-CoV-2 variants to the Spike protein presented on VLPs by ELISA. Each value is the mean ± SD from 3 independent measurements. (Β) Inhibition of RBD binding to the ACE2 receptor by COVA2-15 and 2E8 mAb variants was determined by a competitive ELISA assay. Data shown are 1 representative out of 2 independent experiments with similar results. (C) Binding kinetics of COVA2-15 and 2E8 mAb variants to RBD were obtained by SPR spectroscopy in multicycle kinetic experiments. An anti-His antibody was immobilized on a CM5 chip, RBD-His was captured (50 response units (RU) for COVA2-15 IgG, SIgA1 and SIgA2; 100 RU for 2E8 SIgA1, SIgA2; 300 RU for 2E8 IgG), and 5 or 6 different concentrations of the respective mAb were injected. The obtained curves were fitted with a 1:1 binding model. Data shown are from 1 experiment representative of at least 2 technical repeats.

The binding of IgG and IgA formats to SARS-CoV-2 was assessed as half-maximal effective concentrations (EC_50_) determined by ELISA assays using virus-like particles displaying Spike protein of either the Wuhan or Delta variant (Figure 3A; Table S6). Monomeric and secretory IgA1 and IgA2 COVA2-15 showed strong binding to the Wuhan variant and was comparable to their IgG counterpart with (IgG/mIgA: EC_50_ ∼0.05 nM; SIgA: EC_50_ ∼0.01 nM). Binding of the parental IgG to the Delta variant was much reduced (∼300-fold, EC_50_ ∼9 nM). This was observed to a lesser extent for mIgA1, mIgA2, and SIgA2 (∼10- to 30-fold), whereas SIgA1 maintained stronger binding capacities to Delta (EC_50_ ∼0.09 nM). In general, binding of the 2E8 mAbs was lower for the Wuhan variant compared to COVA2-15 mAbs and 2E8 mAbs binding was less reduced for the Delta variant. Here, an up to 100-fold increase in binding to both virus-like particle (VLP) variants was observed for SIgA1 and SIgA2 compared to monomeric Ig formats (IgG/mIgA: EC_50_ ∼1–2 nM; SIgA: EC_50_ ∼0.02 nM).

In a competitive ELISA assay, COVA2-15 and 2E8 IgG and IgA mAbs were further analyzed for their capability to inhibit RBD binding to the angiotensin-converting enzyme 2 (ACE2) receptor (Figure 3B). Plant-produced IgG and IgA antibodies were able to inhibit RBD binding to ACE2-Fc using this assay, although 2E8 variants needed to be administered in higher molar ratios. In general, secretory IgAs performed better compared to monomeric IgA and IgG as expected due to their multivalency.

The binding kinetics of IgG and monomeric and secretory IgA variants of COVA2-15 and 2E8 to RBD were investigated further using surface plasmon resonance (SPR) spectroscopy. RBD was captured with a CM5 chip with immobilized anti-His antibody, different concentrations of each mAb were injected in multicycle kinetic experiments, and curves were fitted in a 1:1 binding model (Figure 3C). A rapid association (K_A_) and very low dissociation rate (K_D_) were characteristic for all COVA2-15 mAb variants, whereas a moderate K_A_ and faster K_D_ rate were observed for 2E8 IgG. Secretory IgA versions, particularly in the case of 2E8, displayed a more rapid K_A_ and a much-reduced K_D_ rate with an up to 10-fold increase in affinity (K_D_) compared to IgG and monomeric IgA (Tables 1 and S7). This avidity effect was not observed so clearly for the COVA2-15 variants, likely due to the already near-irreversible nature of the interaction of these monomeric formats with RBD.

**Table 1:** Kinetic parameters of COVA2-15 and 2E8 IgG/IgA mAbs to RBD

	*k*_a_, 1/Ms	*k*_d_, 1/s	*K*_D_, nM
COVA2-15 IgG	332,930.6 ± 45,451.2	0.00024 ± 0.00001	0.74 ± 0.08
COVA2-15 SIgA1	422,888.0 ± 66,027.9	0.00016 ± 0.00001	0.38 ± 0.02
COVA2-15 SIgA2	326,233.2 ± 91,502.2	0.00024 ± 0.00003	0.76 ± 0.12
2E8 IgG	70,974.3 ± 12,795.4	0.00543 ± 0.00080	76.93 ± 2.57
2E8 SIgA1	111,255.2 ± 137.2	0.00147 ± 0.00000	13.23 ± 0.02
2E8 SIgA2	155,903.3 ± 39,703.3	0.00155 ± 0.00003	10.56 ± 2.79

Rate constants were determined at 5 different concentrations using a 1:1 binding model. Values are shown as mean ± SD of 2 technical repeats.

### Neutralization activity of different antibody formats

**Figure 4:**
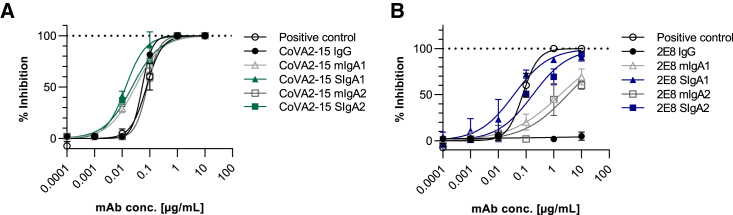
Neutralization of SARS-CoV-2 (England 02/2020) (A and B) Neutralization by COVA2-15 (A) and 2E8 (B) mAbs variants. Neutralization capacity was measured using a plaque reduction neutralization test (PRNT) assay on Vero E6 cells. mAbs were added in serial 1:10 dilutions starting with 10 μg/mL. A positive control (WHO International Standard of anti-SARS-CoV-2 immunoglobulin, 20/136, NIBSC) was included. The mean of duplicates of 1 representative out of 2 experiments with similar results is shown.

The neutralization ability of COVA2-15 and 2E8 IgG and IgA antibodies was investigated using a live virus neutralization assay with a clinical isolate of SARS-COV-2 (England/2/2020) propagated in Vero E6 cells stably expressing ACE2 and transmembrane serine protease 2 (TMPRSS2). Plaques were counted and expressed as percentage of non-neutralizing control (Figure 4; Table S8). All of the COVA2-15 mAb variants showed high neutralization potential with 50% inhibitory dose (ID_50_) values ranging from 13.5 ng/mL for SIgA formats to 50 ng/mL for monomeric IgA and IgG, which are slightly higher compared to previously reported data of COVA2-15 IgG variants produced in a mammalian expression system using different assays (∼8–10 ng/mL).^33^

The RBD-targeting 2E8 mAbs showed a reduced capability for blocking RBD binding to ACE2 in the competition ELISA, suggesting a reduced virus neutralization potency. The IgG version of 2E8 exhibited no inhibition at the tested concentrations, which were below the inhibitory concentrations determined in a previous study.^32^ Monomeric IgA1 and IgA2 were weak neutralizers (ID_50_ ∼1.7–3.2 μg/mL) and only the secretory IgA variants (2E8 SIgA1 ID_50_ ∼36 ng/mL, 2E8 SIgA2 ID_50_ ∼176 ng/mL) had neutralizing activity (Figure 4B).

### Efficacy of intranasally administered anti-SARS-CoV-2 mucosal antibodies in ACE2 transgenic mice

**Figure 5:**
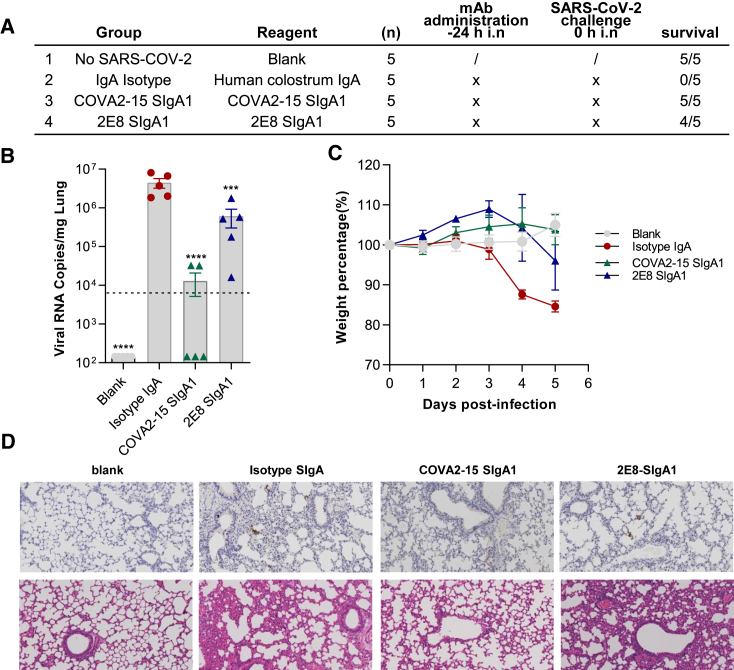
Efficacy of intranasally administered COVA2-15 SIgA1 and 2E8 SIgA1 in hACE2 mice (A) Experimental schedule of mAbs in the prevention and treatment of SARS-CoV-2 infection. The table is a summary of groups (n = 5 mice) with different treatment. Survival rates of all 4 groups were recorded and calculated. (B) Viral loads in lung among 4 groups were measured by qRT-PCR. The name of each group in the x axis is indicated according to the table in (A). Each dot represents 1 mouse. The limit of detection was 2.3 × 10^4^ copies/mg referenced to blank control, which was not infected with SARS-CoV-2 (Blank group). Data represent mean ± SEM. One-way ANOVA was performed to compare treatment groups with the isotype control group. ∗∗p < 0.01; ∗∗∗p < 0.001; ∗∗∗∗p < 0.0001. (C) Body weights of mice among the above 4 groups were recorded. Each line represents data from 1 group. (D) Representative sections of lung were visualized under the 20× objective. H&E staining was conducted to analyze the lung inflammation and observed at 64-fold magnification.

To assess the prophylactic efficacy of COVA2-15 SIgA1 and 2E8 SIgA1 *in vivo*, mAbs were administrated intranasally to human (h)ACE2 transgenic mice 24 h before challenge with SARS-CoV-2 (Figure 5A). This model was previously developed and optimized to assess anti-SARS-CoV-2 IgG mAb-mediated protection. High levels of viral RNA (4.5 × 10^6^ copies/mg) were detected in the lungs of isotype-treated control mice, but significantly reduced in both groups treated with 250 μg (average of 10 mg/kg) COVA2-15 SIgA1 and 2E8 SIgA1 as evidenced by real-time PCR (Figure 5B). Mice receiving COVA2-15 and 2E8 mAbs treatment showed less weight loss than the controls (Figure 5C). The results correlate with clinical protection, with partial protection afforded by 2E8 SIgA1, and with full protection by COVA2-15 SIgA1 (Figure 5A). Histopathological analysis of lung tissues demonstrated that SARS-CoV-2 induced lung lesions, with focal infiltration of inflammatory cells around bronchi and blood vessels and alveolar septal thickening in the control mice. There was also narrowing and collapse of the alveolar wall with the creation of larger cystic cavities. The COVA2-15 SIgA1-treated group exhibited the least pathological changes, whereas the 2E8 SIgA1-treated group histology resembled that of the PBS-treated control group (Figure 5D).

### Aerosolization of IgG and SIgA1 mAbs with a mesh nebulizer

**Figure 6:**
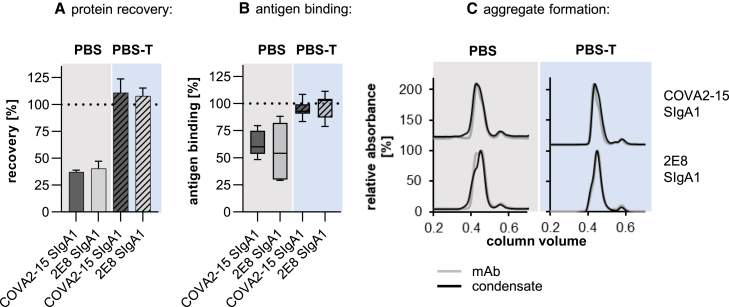
Aerosolization of COVA2-15 and 2E8 SIgA1 antibodies using the Omron MicroAir U22 portable mesh nebulizer; mAb, 1 mL, with a concentration of 100 μg/mL was nebulized and condensate was collected (A) Protein concentration in the condensate after aerosolization was measured using absorbance at 280 nm. (B) Antigen binding of samples before and after nebulization was tested by ELISA with equal protein loading. Values for recovery and antigen-binding activity are means ± SDs of at least 3 independent aerosolization experiments. (C) Overlay of representative size-exclusion chromatograms of mAbs before and after nebulization. Curves are representatives out of 3 repeats with similar results. A total of 200 μg of SIgA1 were injected onto a Superdex 200 Increase 10/300 GL column.

Anticipating the topical delivery of mAbs to the upper and lower respiratory tracts, we explored the use of aerosolization to deliver COVA2-15 and 2E8 SIgA1 mAbs using the widely available Omron MicroAir nebulizer (Omron, UK). Antibodies are bioactive, large molecules with the multimeric structure of SIgA adding additional complexity. The process of aerosolization in a nebulizer may lead to the loss of protein and/or activity. After the aerosolization of SIgA1 in PBS, only 40% of total protein was recovered (Figure 6A). This was associated with a significant loss of antigen-binding capacity (Figure 6B), although no significant amounts of aggregates were detected in the condensate using SEC (Figure 6C). These losses could be reversed by adding 0.05% Tween 20 (Polysorbate-20) to the antibody preparation. Furthermore, increasing the concentration of COVA2-15 SIgA1 in the aerosolization formulation from 100 to 500 μg/mL did not influence recovery. The formation of subvisible aggregates and size distribution of COVA2-15 SIgA1 before and after nebulization was determined using dynamic light scattering (DLS; Figure S6). The average size of 15 nm is in the expected range, with no significant amount of HMWAs in the condensate. The polydispersity index was 0.29 in both samples, suggesting some degree of multidispersity also represented in the broad band in SDS-PAGEs of SIgA and the presence of dimers (molecular weight 469 kDa) as well as tetramers (molecular weight 958 kDa) as shown by SEC light-scattering (SEC-LS). The presence of tetramers is more pronounced for COVA2-15 SIgA1 compared to 2E8 SIgA1, and no shift toward higher oligomers could be observed upon aerosolization (Figure S7). These findings suggest that the aerosolized delivery of SIgA1 using the Omron nebulizer is feasible provided that a nonionic detergent is included in the formulation, although there is further scope for optimization.

## Discussion

In this study, we used the plant-based *N. benthamiana* ΔXT/FT expression platform to produce neutralizing mucosal IgA antibodies against SARS-CoV-2, performed a detailed biochemical and functional analysis of the recombinant antibodies, and explored their potential use to prevent infection in an *in vivo* model. We also addressed two key product development steps by demonstrating that expression levels could be reproduced at pilot scale by a third-party contract manufacturer and that SIgA antibodies can be formulated in a way that makes delivery by aerosol feasible.

All of the monomeric IgG and IgA antibody variants expressed well in plants and were functional in terms of antigen binding. However, the capacity for assembly into multimeric secretory IgA differed between antibodies with different variable regions. mAb COVA2-15 SIgAs displayed almost full assembly (80%–90%) and 2E8 variants demonstrated up to 70% assembly into multimers, thus exceeding yields and ratios of recombinantly produced SIgA to monomeric IgA in previous reports using plant- and mammalian-based expression.^30^^,^^38^^,^^39^^,^^40^^,^^41^ SIgA antibodies based on mAbs 2–15 and COVA1-22 showed very poor assembly into multimers even though they differ from COVA2-15 and 2E8 only in the variable domain sequences. In previous studies, it was suggested that the JC incorporation is the limiting factor for secretory IgA formation.^29^^,^^30^^,^^39^ Other factors that were reported to contribute to dimer formation were the involvement of certain human chaperones, including ERp44 or MZB.1, certain structural features of the CH3 domains of IgA1 and IgA2, and tailpiece glycosylation.^29^^,^^30^^,^^42^ Our data indicate that there may be additional factors contributing to JC incorporation and IgA dimerization that need to be investigated.

COVA2-15 and 2E8, which displayed the highest IgA yield and assembly capacity, were selected for detailed characterization. Class switching of COVA2-15 and 2E8 IgG to monomeric IgA did not significantly influence EC_50_ for binding to the SARS-CoV-2 Spike protein presented on VLPs. However, secretory IgAs displayed 10- to 100-fold increased binding capacities compared to monomeric Igs. These avidity effects can be attributed to the multivalency of SIgA and are particularly prominent when the specific Fab–antigen interaction displays lower affinities. These effects were also apparent in SPR kinetic experiments using recombinant RBD-His. The dissociation rate and consequently the K_D_ of 2E8 SIgA compared to the moderately binding IgG counterpart were much improved, whereas COVA2-15 SIgA behaved similar to the corresponding strong binding IgG.

Strong binding to RBD and competition with the ACE2 receptor binding translated into potent virus neutralization capacities of all of the COVA2-15 mAb variants, with a clear increase (∼10-fold) from monomeric to multimeric antibody formats. The opposite was observed for 2E8-based antibodies, which showed moderate binding to RBD and reduced competition with ACE2 binding compared with COVA2-15. Notably, the two mAbs recognized different epitopes on the ACE2 binding of RBD.^32^^,^^33^ The monomeric Ig formats of 2E8 displayed little or moderate virus neutralization, but the multimeric SIgA formats showed strong inhibition. The increased activity of monomeric IgA over IgG may result from the extended hinge region in IgA1 or other structural differences of the antibody classes. Furthermore, IgA and multimeric antibody formats could enhance the inhibition of virus entry through other mechanisms such as steric hindrance or increased avidity, potentially offering a means of rescuing or repurposing relatively poorly performing IgG antibodies.^40^^,^^43^

SIgA is believed to have a longer half-life in mucosal secretions partly due to unique structural features, making it less susceptible to proteolysis. Consistent with previous observations of an increased half-life for IgA in the mucosa, plant-produced COVA2-15 and 2E8 SIgA variants were significantly slower to degrade in saliva compared to their IgG counterparts.^27^

SIgA also has unique interactions with structural and functional components of the mucosa, which likely contributes to this enhanced stability. Diffusion of SIgA is reduced in mucus gels, a phenomenon called mucus trapping, which is likely conferred by glycan–glycan interactions of the extensive *N*-glycosylation of heavy chains and SCs of IgA and mucus.^44^^,^^45^ In humans, SIgA carries mostly branched complex *N*-glycans with high levels of sialic acid on seven putative glycosylation sites occupied in varying degrees.^46^ This study confirmed that plants performed these complex posttranslational modifications with relatively high homogeneity compared to mammalian production systems and carry *N*-glycans lacking modifications such as β1,2-xylose and core α1,3-fucose, which are commonly found in plants that have not been glycoengineered.^35^^,^^47^ The elongated hinge region of plant-produced IgA1 exhibits plant-specific conversion of prolines to hydroxyprolines, followed by the addition of unbranched arabinose chains, associated with *O*-glycosylation. The potential functional significance of this needs to be studied further, but an earlier study demonstrated that the repeated application of a plant-made SIgA to the human oral cavity did not cause any side effects.^22^ SIgA produced in glycoengineered plants was able to interact with human saliva and commercial mucus preparations.

Neutralizing antibodies against SARS-CoV-2 have been used increasingly in the early treatment of moderate and severe COVID-19 with moderate efficacy, but only administered by the systemic route.^48^ SIgAs applied topically to mucosal sites may provide a different, much earlier intervention to tackle viral carriage and transmission. SARS-CoV-2 is present mainly in the nasopharynx and lungs, so direct administration to the upper respiratory tract may provide faster and more robust antiviral activity in the sites where the virus resides and replicates.^49^^,^^50^

SIgA can, like IgG, directly neutralize virus, and increased valency usually confers increased neutralization capacity, as seen here in the case of 2E8. An important alternative protective mechanism of SIgA in the mucosa is immune exclusion by which SIgA blocks microorganisms and viruses from attaching to mucosal target epithelial cells.^37^^,^^51^ This involves agglutination through antibody-mediated crosslinking, entrapment in mucus via interaction with IgA heavy-chain and SC-linked glycans, and clearance through peristalsis.^14^^,^^52^^,^^53^ Through the tetravalent binding of SIgA resulting in increased avidity and neutralization capacities *in vitro*, as well as interactions with components of the mucosa and saliva that have been demonstrated, it is expected that recombinant plant-produced anti-SARS-CoV-2 SIgA mAbs will act primarily as noninflammatory antibodies through immune exclusion. Furthermore, other mechanisms of actions as well as interactions with other receptors and lectins present on epithelial cells may contribute.^54^

The *in vivo* study addressed protection against SARS-CoV-2 challenge in an hACE2 mouse model that was previously successfully used to demonstrate IgG-mediated protection.^55^^,^^56^^,^^57^^,^^58^^,^^59^^,^^60^ Although this model is appropriate for demonstrating antibody-mediated protection from COVA2-15 and 2E8, the differences between the oral mucosal environment in mice and humans are such that we would not expect that this model could be used to illustrate any of the nuanced differences between different types of human antibodies.^61^^,^^62^ We believe that the functional advantages of human SIgA mAbs are likely only to be discernible *in vivo*, at human clinical trials, in the context of a human mucosal environment and microbiome.

Systemically delivered mAbs will undoubtedly continue to be used, but they could be replaced by regular topical delivery. This would have the advantage that high local doses delivered by aerosol droplets or particles, with limited systemic exposure, can achieve therapeutic equivalency to much higher doses delivered systemically. Although nasal spray is the delivery mode of choice in a nonclinical setting because it can be easily formulated and distributed, aerosolization for delivery to the upper and lower respiratory tract may be required in clinical settings. We performed initial studies to test the feasibility of this concept for SIgA1. We demonstrated that with the addition of 0.05% Tween 20 to the antibody formulation, protein recovery and antigen-binding activity can largely be recovered from the condensate. Tween 20 is permissible for human application in low concentrations and is used in foods and medications as a dispersant or an excipient.^63^^,^^64^

In summary, we demonstrated that neutralizing IgA mAbs against SARS-CoV-2 can be produced as monomeric and secretory formats in a plant-based expression system at high yields, as well as in a pilot-scale production setting. We showed that the secretory IgA antibodies are able to maintain their structure and binding affinities when incubated in the harsh environment of human saliva. More important, we showed that these plant-generated antibodies have strong virus neutralization activity and can reduce SARS-CoV-2 infection in an *in vivo* murine model. Our preliminary data provide a strong case for the development of secretory IgAs as prophylaxis/postexposure prophylaxis and/or clinical management of COVID-19 and other respiratory infections.

## Materials and methods

### Construct design and cloning

*N. benthamiana* codon-optimized sequences of the heavy- and light-chain variable regions of COVA2-15 (GenBank: QKQ15273.1, QKQ15189.1), COVA1-22 (GenBank: QKQ15169.1, QKQ15253.1), 2–15 (PDB: 7L57_H, 7L57_L), and 2E8 IgG mAbs flanked with BsaI type II restriction sites were synthesized by GeneArt (Thermo Fisher Scientific).^31^^,^^33^ Using Golden Gate assembly, the variable heavy-chain sequences were cloned into Gateway pDONR-based plasmids (Invitrogen) between a human Ig heavy-chain leader sequence (MELGLSWIFLLAILKGVQC) and either human gamma-1 (GenBank: AAA02914.1), alpha-1 (GenBank: AAT74070.1), or alpha-2m(1) (GenBank: AAT74071.1) constant regions. Variable light-chain fragments were inserted between the human light-chain leader sequence (MDMRVPAQLLGLLLLWLPGARC) and either human kappa constant regions for COVA2-15 variants (GenBank: AAA58989.1) or lambda constant regions for COVA1-22, 2–15, and 2E8 variants (GenBank: CAA40940.1).^21^ Full-length heavy- and light-chain genes were separately subcloned into the binary high expression vector pEAQ-HT-DEST3 using Gateway cloning.^65^ Human SC and JC constructs cloned separately into pEAQ-HT have been described previously.^66^ The pEAQ-HT plant expression vectors containing the gamma and alpha heavy chains as well as the kappa and lambda light chains were transformed into *Agrobacterium tumefaciens* strain GV3101 (Leibniz Institut DSMZ-Deutsche Sammlung von Mikroorganismen und Zellkulturen GmbH, DSM 12364) by electroporation.

The construct for expression of the RBD of the SARS-CoV-2 Spike (PDB: 6VYB, R319-F541) with a C-terminal 6xHis-tag cloned into pCAGGS (NovoPro, China) mammalian expression vector was provided by Mark Dürkop from the University of Natural Resources and Life Sciences, Vienna, Austria.

### Transient expression of IgG and IgA variants in *N. benthamiana*

*Agrobacteria* containing the appropriate constructs were grown overnight at 28°C in lysogeny broth containing 25 μg/mL rifampicin and 50 μg/mL kanamycin. For the expression of IgG or monomeric IgA1 and IgA2 variants, the overnight cultures containing the respective constructs for the heavy and light chains were diluted in infiltration buffer (10 mM morpholinoethanesulfonic acid, 10 mM MgSO_4_, and 0.1 mM acetosyringone) to an optical density 600 (OD_600_) of 0.1. For secretory IgA variants, heavy- and light-chain constructs were diluted to an OD_600_ of 0.05 and mixed with the JC construct at an OD_600_ of 0.2 and the SC construct at an OD_600_ of 0.1. *Agrobacteria* solutions were then introduced into 6- to 8-week-old glycoengineered *N. benthamiana* ΔXT/FT plants by vacuum infiltration.^34^^,^^67^ Plants were grown in a controlled environment room at 25°C with an 16/8-h light/dark cycle. After 5 days, infiltrated leaf material was harvested, and crude leaf extract was prepared by adding 3 volumes of ice-cold PBS pH 7.4 containing 0.1% (v/v) Tween 20 in a blender. Homogenized leaf material was passed through a Miracloth filter (Merck Millipore, Germany) and centrifuged at 20,000 × *g* for 1 h, followed by filtration through 0.45-μm pore size filters (Durapore membrane filter, Merck Millipore).

### Purification of IgG and IgA variants from crude leaf extract

Clarified leaf extracts were passed through columns packed with either Pierce Protein A resin for purification of IgG and COVA2-15 IgA variants or CaptureSelect IgA affinity matrix (both Thermo Fisher Scientific) for purification of 2E8 IgA variants equilibrated with PBS. Proteins were eluted with 0.1 M glycine pH 2.7, followed by the immediate addition of 10% (v/v) 1 M Tris-HCl pH 9.0 to neutralize the pH. Fractions containing the protein of interest were pooled and dialyzed against PBS at 4°C overnight using a dialyzing cassette with 10-kDa molecular weight cutoff (MWCO; Slide-A-Lyzer, Thermo Scientific, USA). Pooled and dialyzed protein fractions were concentrated using Amicon centrifugal filters with an MWCO of 100 kDa (Merck Millipore) and subjected to SEC on a HiLoad 16/600 Superdex 200 pg column (GE Healthcare, USA) equilibrated with PBS pH 7.4 connected to an ÄKTA pure (GE Healthcare, USA) fast protein LC system.

### Expression and purification of RBD-His

For production of the recombinant receptor binding domain of the SARS-CoV-2 (Wuhan) Spike protein, Expi293F cells were maintained and transfected according to the manufacturer’s manual in FreeStyle expression medium (all Thermo Fisher Scientific). High-quality plasmid preparations were obtained using a Plasmid Midi kit (Qiagen, USA). For the transfection of 200-mL culture with a cell density of 3.0 × 10^6^ cells/mL, a total of 200 μg plasmid DNA was mixed in 4 mL OptiPro SFM medium and combined with another 4 mL OptiPro containing 640 μL ExpiFectamin (all Thermo Fisher Scientific) before gradual introduction to the cells. The culture was incubated for 7 days at 37°C in a humidified atmosphere with 8% CO_2_ on an orbital shaker rotating at 125 rpm. The was harvested by centrifugation at 20,000 × *g* for 30 min at 4°C and additionally filtrated through a 0.45-μm Durapore membrane filter (Merck Millipore). Clarified cell supernatant was diluted 1:2 in loading buffer (20 mM Tris, 500 mM NaCl, and 10 mM imidazole). The solution was loaded onto a 5-mL HisTrap HP column (GE Healthcare) equilibrated with 5 column volumes of loading buffer, and bound protein was eluted by applying buffer containing 20 mM Tris, 500 mM NaCl, and 300 mM imidazole. Fractions containing the protein of interest were pooled and dialyzed against PBS at 4°C overnight using a dialyzing cassette with an MWCO of 10 kDa (Slide-A-Lyzer). Pooled and dialyzed protein fractions were concentrated using Amicon centrifugal filters with an MWCO of 100 kDa (Merck Millipore).

### SDS-PAGE

A total of 5 μg of purified mAbs were resolved on a NuPage 4%–12% Bis/Tris gel (Life Technologies, UK) and stained with InstantBlue (Expedeon, UK).

### ELISA

For the quantification of IgG and IgA mAbs in clarified crude extract of infiltrated *N. benthamiana* plants, ELISA plates were coated with 250 ng/well anti-human gamma chain antiserum (AU004, Binding Site, UK) and goat polyclonal antibody to human anti-alpha chain (ab97211, Abcam, UK) in PBS pH 7.4 at 4°C overnight, respectively. After blocking with PBS containing 2% (w/v) BSA and 0.1% Tween 20 (v/v), clarified crude plant extracts were added to the wells in normalized concentrations and incubated for 1.5 h at 37°C. As standards, IgG1/kappa or IgG1/lambda isolated from human myeloma plasma (15154, I5029, Sigma, USA), purified human IgA (P80-102, Bethyl, USA), and IgA from human colostrum (I2363, Sigma) were used. The detection of secretory IgA variants was carried out with mouse anti-SC antibody (SAB4200787, Sigma), followed by HRP-labeled anti-mouse antibody (SAB5300168, Sigma). For IgG and monomeric IgA variants, HRP-labeled anti-kappa (A18853, Invitrogen, USA) or anti-lambda light-chain (ab200966, Abcam) antisera were used. After incubation for 1 h at 37°C, plates were developed using 3,3′,5,5′-tetramethylbenzidine (Thermo Fisher Scientific) substrate, the reaction was stopped with 2 M H_2_SO_4_, and the readout was performed on an Infinite F200 Pro plate reader (Tecan, Switzerland) at a 450-nm wavelength.

For determination of the ratio of functional and fully assembled SIgA to total IgA in each size-exclusion fraction, similar ELISA assays were performed. Capture was with 100 ng/well purified recombinant RBD-His or anti-alpha HC antibody (ab97211, Abcam). Purified mAbs were diluted to 2 μg/mL in blocking solution and added to RBD and anti-IgA-coated plates in normalized concentrations and incubated for 1.5 h at 37°C. The detection of SC or antibody kappa or lambda chains was carried out as described above.

To determine the binding of the purified recombinant mAbs to SARS-CoV-2 RBD, ELISA plates coated with either 100 ng/well purified RBD-His, 100 ng/well purified trimeric Spike (kind gift from Dr. Svend Kjaer, The Francis Crick Institute), or 250 ng/mL purified plant-produced VLPs containing Wuhan-Hu-1 S protein (OK413878) or Delta variant S protein (OM858819) were used.^68^ The purified mAbs were added to the wells in normalized concentration. For detection, HRP-labeled anti-human kappa or lambda light-chain antibodies were used as above. The EC_50_ was calculated in GraphPad Prism 9.0.

### Competitive ELISA

To determine the capability of purified mAbs to inhibit the binding of RBD-His to the ACE2 receptor, a competitive binding ELISA was performed. Purified ACE2-Fc was kindly provided by Elisabeth Lobner (University of Natural Resources and Life Sciences, Vienna), and 500 ng/well were coated on an ELISA plate at 4°C overnight, followed by blocking with PBS containing 2% (w/v) BSA and 0.1% Tween 20 (v/v). RBD-His was incubated with varying molar ratios of the different mAbs starting with a 2:1 [mAbs:RBD-His] that was stepwise reduced to 0.007:1 for 1 h at 37°C before addition to the wells. Binding of RBD-His to ACE2-Fc was detected using an HRP-labeled anti-His antibody (71840, Sigma), and plates were developed as described above.

### SPR spectroscopy

The binding kinetics of plant-produced IgG and IgA mAbs to SARS-CoV-2 RBD-His were determined on a BIAcore X-100 instrument (GE Healthcare) at 25°C with HBS-EP+ buffer (10 mm HEPES pH 7.4, 150 mM NaCl, 3 mM EDTA, and 0.05% surfactant P-20). The monoclonal mouse anti-His antibody (SAB2702220, Sigma) was immobilized onto a CM5 chip with standard amine coupling. Purified RBD-His was diluted in HBS-EP+ buffer and injected at a concentration of 1 μg/mL for 30 s at a flow rate of 30 μL/min, followed by the injection of 5 different concentration of each mAb with a flow rate of 30 μL/min. The second-lowest concentration was repeated to ensure reproducibility and allowed to dissociate before regeneration with 10 mm glycine pH 1.7 for 1 min at the flow rate of 10 μL/min. Referenced and blanked sensorgrams were fitted with BIAcore Evaluation software using a 1:1 Langmuir model of binding. Each assay was performed in duplicate.

### MS

A total of 20 μg purified protein was reduced, S-alkylated, and digested with trypsin (Promega, USA). Glycopeptides were then analyzed by capillary reversed-phase chromatography and electron-spray MS using an Agilent Series 6560 LC-IMS-QTOFMS instrument as reported previously.^35^

### mAb stability assays in human saliva

Saliva was donated by two healthy volunteers and processed immediately after collection. Neither donor had knowingly had a previous natural infection with SARS-CoV-2 but both had received a two-dose vaccination regime and their salivas had been shown to contain low levels of RBD-specific IgG but not SIgA antibodies (data not shown). The saliva was clarified by centrifugation at 3,000 × *g* for 15 min. Supernatants were collected and aliquoted into 100-μL aliquots before being mixed with 10 μg of each IgG and SIgA mAb variant in a volume <5 μL. Following the immediate collection of a 15-μL sample (0 min time point), antibody/saliva solutions were incubated at 37°C and sampled at 5, 60, 150, 240, and 1,440 min. Samples were analyzed using a sandwich ELISA assay as described above, using plates coated with 100 ng/well purified recombinant RBD-His in PBS pH 7.4. The mAbs/saliva solutions were diluted in blocking buffer 1:1,000, added to the wells in normalized concentrations and incubated for 1.5 h at 37°C. The corresponding purified mAb in PBS buffer with known concentration was used as control. IgG and SIgA mAbs were detected using HRP-labeled mouse anti-IgG-Fc (AP113P, Merck Millipore) and mouse anti-SC (IgA) antibody (SAB4200787, Sigma), followed by HRP-labeled anti-mouse antibody (SAB5300168, Sigma), respectively.

### Fast microequilibration dialysis

A 1% (w/v) solution of MUCII from porcine stomach (Sigma-Aldrich) was prepared in 1× PBS pH 7.4. Saliva samples were treated as described above. Purified mAbs were mixed with either 50 μL of 1× PBS pH 7.4, MUCII, or saliva to a final concentration of 1 μg/μL. Samples were placed into a fast microequilibration dialysis device and dialyzed overnight at 4°C against 50 μL 1× PBS pH 7.4 using 0.05-μm polycarbonate membranes (both Harvard Bioscience, USA). The concentration of mAbs in pre- and postdialyzed samples was determined using the same antigen sandwich ELISA format from the previous section.

### Virus neutralization assay

Vero E6 cells stably expressing ACE2 and TMPRSS2 were obtained by the UK National Institute for Biological Standards and Control (NIBSC) and grown in DMEM supplemented with 10% heat-inactivated fetal calf serum (FCS; Gibco, Thermo Fisher Scientific), penicillin (100 U/mL, Sigma), streptomycin (100 μg/mL, Sigma), hygromycin B (250 μg/mL, Thermo Fisher Scientific), and G418 (250 μg/mL, Thermo Fisher Scientific). For plaque reduction assays, cells were seeded to obtain confluent monolayers (10^5^ cells/well in 12-well plates) and allowed to settle overnight. Monolayers were visually inspected before use.

SARS-CoV-2 (England/2/2020) was obtained from Public Health England and passaged in Vero E6 cells stably expressing ACE2 and TMPRSS2. Virus stocks were quantified with a standard plaque assay and expressed as plaque-forming units (PFU) per milliliter.

Purified mAbs were serially diluted 10-fold starting at 15–20 μg/mL in DMEM with 2% FCS and incubated for 1 h at 37°C with 30–40 PFU of SARS-CoV-2 (isolate England/2/2020). After incubation, the virus–antibody mixture was transferred onto a confluent monolayer of Vero-E6 cells expressing ACE2 and TMPRSS2 (NIBSC). After 60 min of adsorption at 37°C, the inoculum was removed and replaced with an overlay containing growth medium (DMEM with 10% FCS) and 0.8% Avicel (Sigma). The monolayers were incubated at 37°C, 5% CO_2_ for 48 h and then fixed and stained with paraformaldehyde 10% (Sigma) and crystal violet (1×, Sigma), respectively. Plaques were counted and expressed as percentage of a neutralizing positive control (World Health Organization [WHO] International Standard for anti-SARS-CoV-2 immunoglobulin, 20/136, NIBSC). The percentage neutralization (inhibition) was calculated in MS Excel and GraphPad Prism 9.0.

### Protective efficacy of COVA2-15 IgG and SIgA in infected hACE2 transgenic mice

A total of 20 eight-week-old male hACE2 transgenic mice (H11-K18-hACE2, no. T037657 GemPharmatech, China) were challenged with 1 × 10^3^ PFU SARS-CoV-2 (CSTR: 16533.06. IVCAS 6.7512). The mice were split into four groups (n = 4) as described in (Figure 5A). Mice without any challenge and treatment served as blank control (blank, n = 5). Mice challenged with SARS-CoV-2 and 250 μg human colostrum IgA (I2636, Sigma, IgA isotype control, n = 4) administered intranasally 24 h before infection served as isotype-treated controls. For the prophylactic group, SIgA1 at a dose of 250 μg/mouse (average of 10 mg/kg) was administered intranasally 24 h before infection (COVA2-15 SIgA1 and 2E8 SIgA1, −24 h, n = 5). The body weight of each mouse was measured daily. The mice were sacrificed 6 days postinfection or at the humane endpoint. Lungs were collected for viral load determination and tissue sections for histopathology. H&E and immunohistochemical (IHC) staining were performed, respectively.

### Viral load measurement by qRT-PCR

Viral RNA was extracted using the TRI Reagent (Sigma). Reverse transcription was performed using HiScript II Q RT SuperMix for qPCR (R223-01, Vazyme, China). Subsequently, 2 μL cDNA was added into a 20-μL qRT-PCR reaction containing the ChamQ SYBR qPCR Master Mix (Q341-02, Vazyme). The primers designed to target the nucleocapsid protein of SARS-CoV-2 were (forward) 5′-GGG GAA CTT CTC CTG CTA GAA T-3′ and (reverse) 5′-CAG ACA TTT TGC TCT CAA GCT G-3′. PCRs were run according to the manufacturer’s instructions. The amount of viral RNA was normalized to the standard curve from a plasmid containing the full-length SARS-CoV-2 *N* gene.

### IHC staining of SARS-CoV-2-infected cells in tissues

Left lung tissues were immersed in 10% neutral buffered formalin (Z2902, Sigma) for 24 h. After the formalin fixation, the tissues were placed in 70% ethanol and subsequently embedded with paraffin. Tissue sections (4-μm thick) were used for IHC staining for SARS-CoV-2 detection using a coronavirus nucleocapsid antibody (40143-MM05, Sino Biological, China). Images were obtained by OLYMPUS IX73 using HCImage Live (×64) software and analyzed by ImageJ (NIH).

### Study approval

All of the animals infected by SARS-CoV-2 were handled in Biosafety Level 3 animal facilities in accordance with the recommendations for care and use of the institutional review board of the Wuhan Institute of Virology of the Chinese Academy of Sciences (Ethics Number: WIVA11202003). All of the authors declare their compliance with publishing ethics.

### Aerosolisation of monoclonal antibodies

COVA2-15 and 2E8 SIgA1 were aerosolized using a commercially available Omron Micro Air U22 electronic mesh nebulizer as previously described.^17^

### DLS

DLS measurements were performed with protein concentrations of 500 μg/mL in 1× PBS pH 7.4 supplemented with 0.1% Tween on a Malvern Zetasizer nano-ZS (Malvern Instruments, UK) in a 12-μL quartz cuvette. Samples were measured at 25.0°C and the LS was detected at 173° and collected in automatic mode. Mean values and SEs of the number weighted diameter were calculated from three measurements for each sample, and each reported value is an average of 12 runs. The resulting data were analyzed using the DTS (version 4.2) software (Malvern Instruments).

### SEC-LS

SEC-LS was used to characterize the recombinant expressed proteins in solutions relating to their purity, native oligomers or aggregates, and molecular weights. Analyses were performed on an OMNISEC multidetector gel permeation chromatography (GPC)/SEC system equipped with a refractive index detector, a right-angle LS detector, a low-angle LS detector and a UV/visible light photodiode array detector (Malvern Panalytical, UK). A Superdex 200 Increase 10/300 GL column (Cytiva, USA) was used and equilibrated with Dulbecco’s PBS without Ca and Mg, P04-361000 (PAN-Biotech, Germany) as running buffer. Experiments were performed at a flow rate of 0.5 mL min^−1^ at 25°C and analyzed using OMNISEC software version 11.40 (Malvern Panalytical). Proper performance of the instrument was ensured by calibration and verification using the 200-μg Pierce BSA standard (Thermo Fisher Scientific). Before analysis, samples were centrifuged (16,000 × *g*, 10 min), and filtered through 0.2-μm Durapore PVDF centrifugal filter(s) (Merck Millipore). A 100-μL volume of each sample was injected, having different concentrations between 0.1 and 0.5 mg/mL.

## Data and code availability

Upon publication, the variable domain gene sequences of the 2E8 antibody will be deposited in the Coronavirus Antibody Database, CoV-Ab-Dab (Oxford Protein Informatics Group) and GenBank. Any additional information required to reanalyze the data reported in this paper is available from the lead contact upon request.

## Supplemental information

Document S1. Figures S1–S7 and Tables S1–S8

Document S2. Article plus supplemental information
