# Supplementary material for: Recombinant neutralizing secretory IgA antibodies for preventing mucosal acquisition and transmission of SARS-CoV-2
Source: Mol Ther. 2024 Jan 24;32(3):689–703. doi: 10.1016/j.ymthe.2024.01.025 (PMC10928148; doi:10.1016/j.ymthe.2024.01.025)
Supplement: Document S1. Figures S1–S7 and Tables S1–S8 [file mmc1.pdf]

YMTHE, Volume *32*

## Supplemental Information

# Recombinant neutralizing secretory IgA antibodies for preventing mucosal acquisition and transmission of SARS-CoV-2

**Kathrin Göritzer, Elisabetta Groppelli, Clemens Grünwald-Gruber, Rudolf Figl, Fengfeng Ni, Huimin Hu, Yuncheng Li, Yalan Liu, Qinxue Hu, Rama Devudu Puligedda, Jae-Wan Jung, Richard Strasser, Scott Dessain, and Julian K.-C. Ma**

## Supplemental Information

## Supplemental Figures

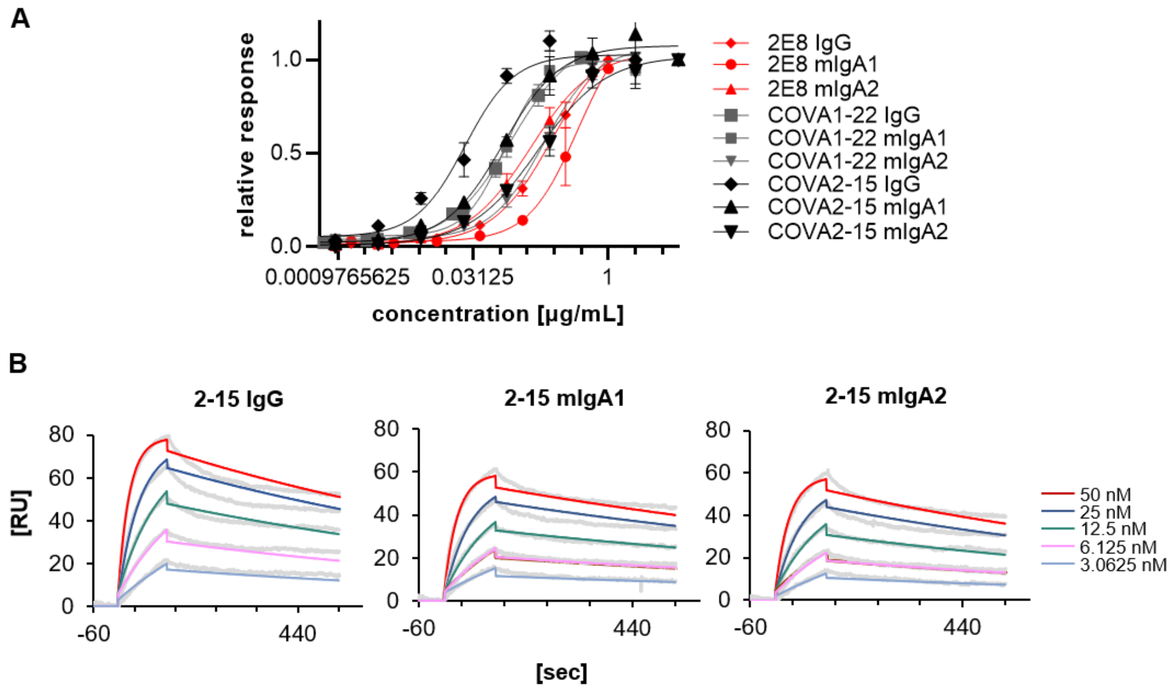


**Figure S1**: **Binding of affinity purified COVA2-15, COVA1-22, 2-15 and 2E8 IgG, monomeric IgA1 and IgA2 to SARS-CoV-2 spike protein.** (**A**) Binding of COVA1-22, 2E8 and COVA2-15 variants to ELISA plates coated with 100 ng/well full-length trimeric spike protein (Wuhan, kind gift from Dr. Svend Kjaer, the Crick Institute). Each displayed value is the mean ± SD from three independent measurements. (**B**) Binding kinetics of 2-15 variants to RBD were obtained by SPR spectroscopy in multi-cycle kinetic experiments. An anti-His antibody was immobilized on an CM5 chip, RBD-His was captured (20 RU), and 5 different concentrations of the respective mAb were injected. The obtained curves were fitted with a 1:1 binding model. Data shown are from one experiment representative of at least two technical repeats. The calculated $K_D$ values are 3.75 nM for 2-15 mIgA1, 2.88 nM for 2-15 mIgA2 and 1.16 nM for 2-15 IgG.

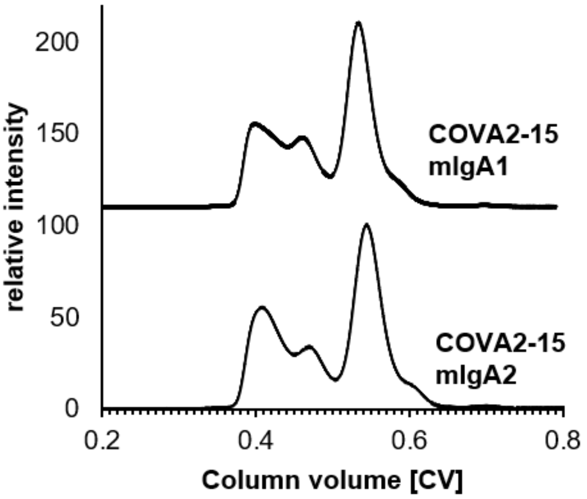


**Figure S2: Normalized size-exclusion chromatograms of affinity-purified monomeric IgA1 and IgA2 variants of COVA2-15 from infiltrated *N. benthamiana* ΔXT/FT leaves**.

Values were normalized based on the highest signal of each chromatogram.

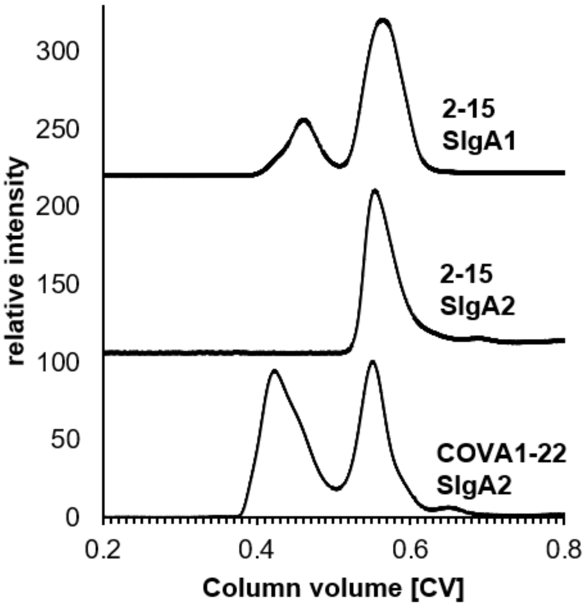


**Figure S3: Normalized size-exclusion chromatograms of affinity-purified 2-15 and COVA1-22 IgA1 and IgA2 which were co-expressed with the joining chain and secretory component in *N. benthamiana* ΔXT/FT leaves to obtain multimeric secretory IgA.** Values were normalized based on the highest signal of each chromatogram

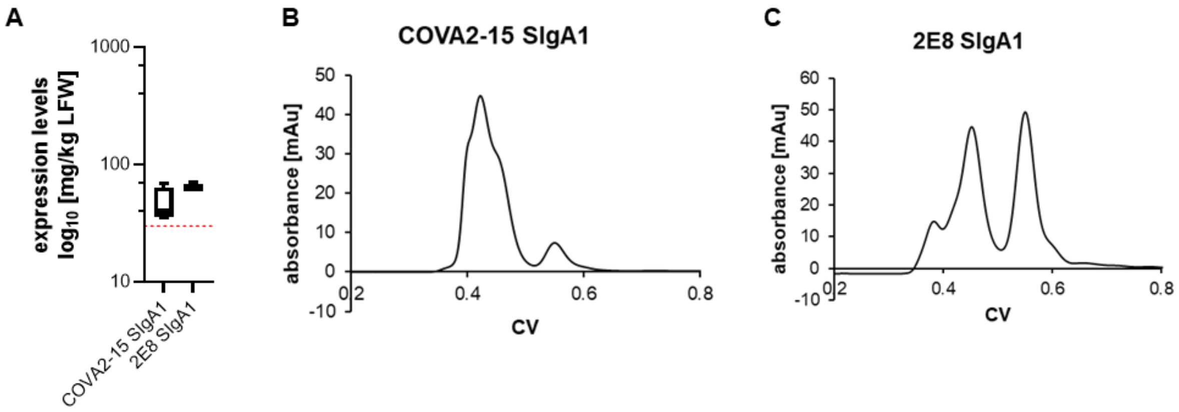


**Figure S4: COVA2-15 and 2E8 secretory IgA1 were transiently expressed in *N.benthamiana* plants in pilot-scale 500 g LFW batches**. **(A)** Expression levels were quantified by sandwich ELISA in crude leaf extracts. SIgA antibodies were detected using anti-secretory component antibodies for all SIgA variants. Quantification data represent the mean of two technical repeats of three independent biological repeats ± SD. Normalized size-exclusion chromatograms of affinity-purified COVA2-15 SIgA1 (**B**) and 2E8 SIgA1 (**C**) from infiltrated *N. benthamiana* ΔXT/FT leaves.

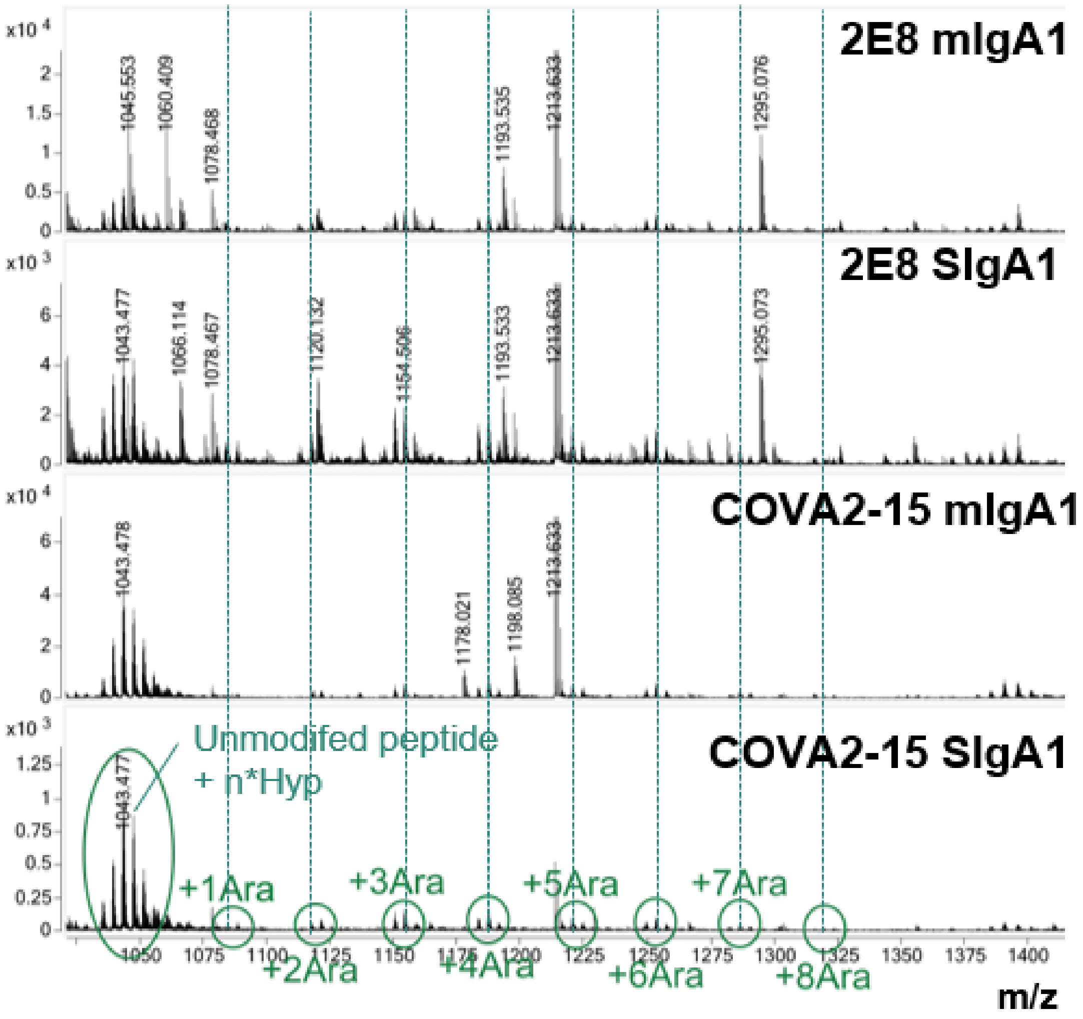


**Figure S5**: **O-glycosylation profiles of *N. benthamiana* ΔXT/FT derived recombinant COVA2-15 and 2E8 monomeric and secretory IgA.** Mass spectra of the hinge region peptide are shown and glycosylated peaks are indicated as arabinoses (Ara) and hydroxyprolines (Hyp).

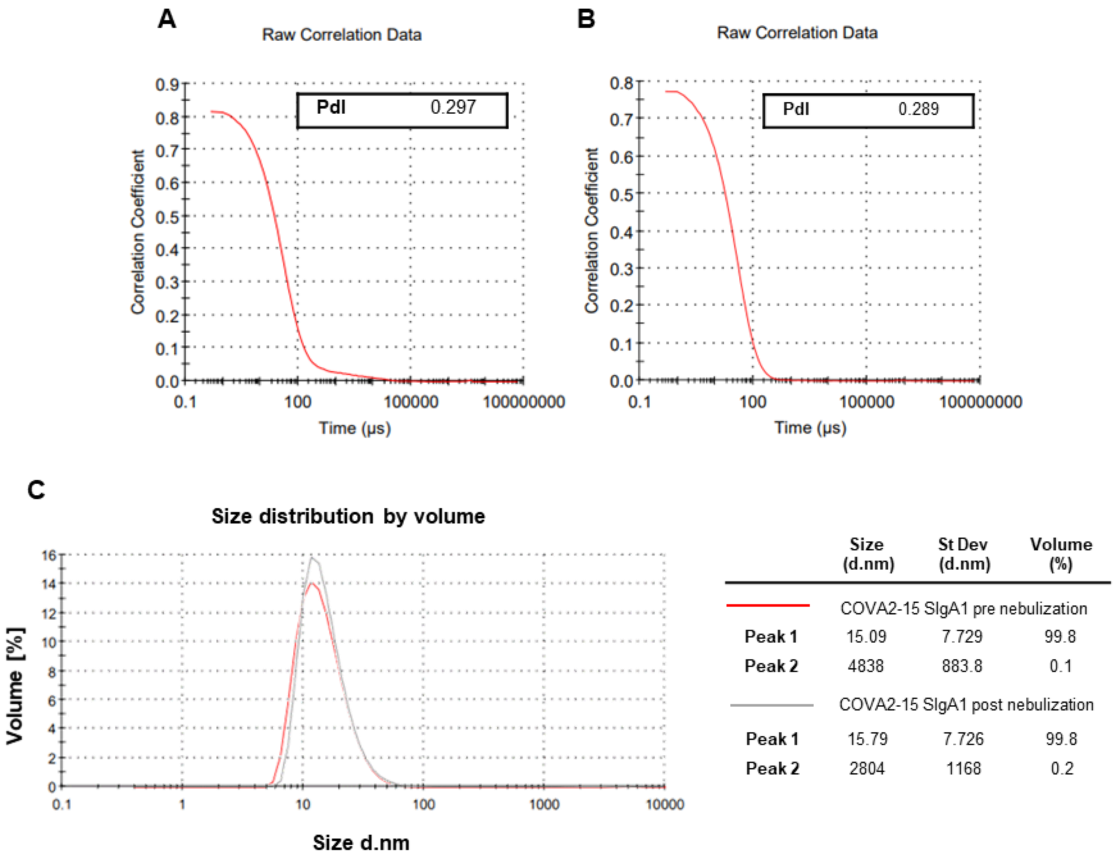


**Figure S6: Dynamic light scattering of COVA2-15 SIgA1 before and after aerosolization using the Omron MicroAir U22 portable mesh nebulizer**. The mean diameter and the homogeneity were measured using a Malvern Zetasizer nano-ZS (Malvern Instruments Ltd., Worcestershire, UK) at 25°C. Each sample was measured in triplicates. The homogeneity of the antibody solution was determined by the polydispersity index (PdI) from the raw correlation before (A) and after (B) nebulisation. Size distribution was calculated by the volume report (C).

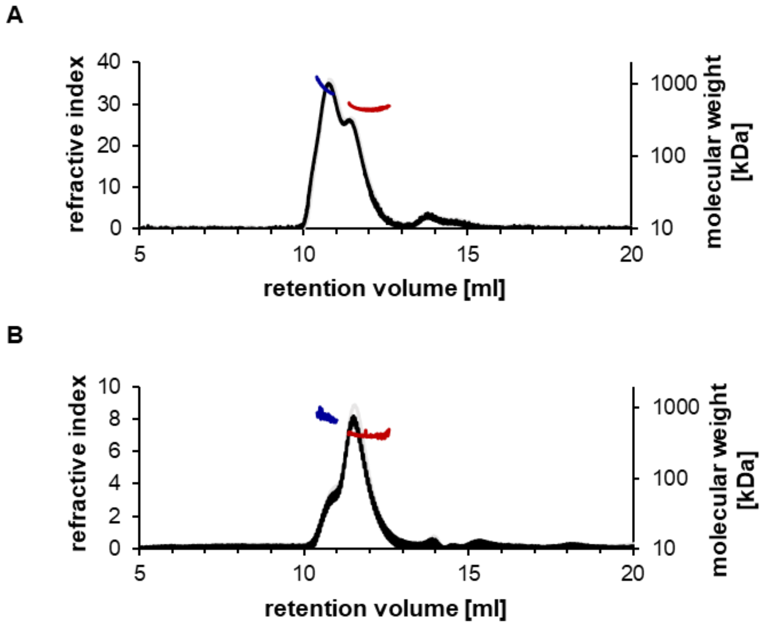


**Figure S7: Size-exclusion chromatography light-scattering of COVA2-15 SIgA1 (A) and 2E8 SIgA1 (B) before and after nebulization**. Overlays of the elution profiles of COVA2-15 SIgA1 (nebulization concentration: 0.5 mg/mL) and 2E8 SIgA1 (nebulization concentration: 0.1 mg/mL) before (grey) and after (black) aerosolization are shown. The depicted molar masses of the tetrameric (blue) and dimeric (red) SIgA species were derived from light-scattering and are as follows in the formulation/the condensate: COVA2-15 SIgA1: 958/974 kDa, 468/470 kDa; 2E8 SIgA1:728/786 kDa, 409/410 kDa.

**Supplemental Tables**

**Table S1: Transient expression levels of monoclonal IgG and IgA antibodies in *N. benthamiana* ΔXT/FT.** IgG, monomeric and secretory IgA1 and IgA2 versions of 4 different mAbs recognizing the SARS-CoV-2 spike proteins were transiently expressed in plants. Expression levels were quantified by sandwich ELISA in crude leaf extracts. Detection of monomeric IgA and IgG variants was with either HRP-labeled anti-kappa (COVA2-15) or anti-lambda light chain (2E8, COVA1-22, 2-15) antibodies. SIgA antibodies were detected using anti-secretory component antibodies for all SIgA variants. Quantification data represent the mean of two technical repeats of three independent biological repeats ± SD.

| mAb | expression levels [mg/kg LFW] | | |
|---|---|---|---|
| COVA2-15 IgG | 134.90 | ± | 30.78 |
| COVA2-15 mIgA1 | 112.50 | ± | 10.12 |
| COVA2-15 mIgA2 | 103.30 | ± | 12.69 |
| COVA2-15 SIgA1 | 33.65 | ± | 5.99 |
| COVA2-15 SIgA2 | 45.49 | ± | 15.90 |
| 2E8 IgG | 100.10 | ± | 17.79 |
| 2E8 mIgA1 | 74.57 | ± | 10.91 |
| 2E8 mIgA2 | 45.40 | ± | 12.65 |
| 2E8 SIgA1 | 35.71 | ± | 8.41 |
| 2E8 SIgA2 | 23.07 | ± | 7.48 |
| 2-15 IgG | 52.48 | ± | 8.89 |
| 2-15 mIgA1 | 74.57 | ± | 10.91 |
| 2-15 mIgA2 | 45.40 | ± | 12.65 |
| 2-15 SIgA1 | 10.03 | ± | 2.95 |
| 2-15 SIgA2 | 1.77 | ± | 0.95 |
| COVA1-22 IgG | 74.84 | ± | 20.00 |
| COVA1-22 mIgA1 | 79.47 | ± | 11.11 |
| COVA1-22 mIgA2 | 10.89 | ± | 2.76 |
| COVA2-15 SIgA2 | 6.36 | ± | 0.41 |
| COVA1-22 SIgA1 | 0.29 | ± | 0.34 |

**Table S2: Purification yields of monoclonal IgG and SIgA antibodies produced in *N. benthamiana* ΔXT/FT after affinity chromatography and size-exclusion chromatography**. Concentration in the pooled and concentrated SEC-fractions were quantified via A280 nm

absorbance. Data represent the mean of three obtained from three independent purifications from 100 g leaf fresh weight ± SD.

| **mAb** | **purification yield [mg/kg LFW]** | | |
|---|---|---|---|
| COVA2-15 IgG | 36.20 | ± | 6.20 |
| COVA2-15 SIgA1 | 25.90 | ± | 0.90 |
| COVA2-15 SIgA2 | 32.00 | ± | 4.00 |
| 2E8 IgG | 38.10 | ± | 8.10 |
| 2E8 SIgA1 | 19.75 | ± | 0.75 |
| 2E8 SIgA2 | 10.20 | ± | 0.60 |

**Table S3: Quantification of the relative abundance (%) of *N*-glycans detected on the heavy chain of IgG and IgA isotypes produced in *N. benthamiana* ΔXT/FT**. *N*-glycans are abbreviated according to the ProGlycAn system (www.proglycan.com). The symbols for the monosaccharides are drawn according to the nomenclature from the Consortium for Functional Glycomics.

| **2E8 mAb** | **mIgA1** | | **SIgA1** | | **mIgA2** | | | | **SIgA2** | | | | **IgG** |
|---|---|---|---|---|---|---|---|---|---|---|---|---|---|
| | NLT | NVS | NLT | NVS | NVT | NLT | NIT | NVS | NVT | NLT | NIT | NVS | NST |
| **non glyc.** | 0.2 | 38.3 | 0.6 | 36.1 | 0.0 | 0.5 | 0.0 | 19.9 | 0.0 | 0.8 | 0.0 | 31.6 | 11.3 |
| GnGn | 12.8 | 3.2 | 7.1 | 3.5 | 14.1 | 6.1 | 7.6 | 4.5 | 18.4 | 4.6 | 11.6 | 5.2 | 60.7 |
| MGn | 23.1 | 3.4 | 15.5 | 4.2 | 18.4 | 15.1 | 21.0 | 3.4 | 16.2 | 10.8 | 21.5 | 3.8 | 22.5 |
| MM | 11.1 | 3.8 | 9.3 | 4.9 | 28.7 | 7.2 | 26.7 | 4.8 | 21.4 | 5.1 | 20.0 | 4.2 | 1.0 |
| GnGnF | 0.0 | 4.1 | 0.0 | 1.0 | 2.6 | 0.0 | 2.5 | 3.7 | 5.9 | 0.0 | 3.0 | 1.1 | 0.7 |
| MGnF | 0.0 | 1.7 | 0.0 | 0.5 | 4.8 | 0.0 | 6.6 | 1.3 | 3.5 | 0.0 | 5.8 | 0.5 | 1.4 |
| MMF | 0.0 | 0.0 | 0.0 | 0.0 | 5.5 | 0.0 | 8.8 | 0.0 | 3.5 | 0.0 | 5.4 | 0.0 | 0.0 |
| Man4 | 2.7 | 2.4 | 3.1 | 2.1 | 2.0 | 2.7 | 3.8 | 2.3 | 2.7 | 2.7 | 3.6 | 1.0 | 0.0 |
| Man5 | 4.1 | 4.3 | 3.4 | 4.2 | 2.3 | 4.5 | 4.1 | 1.6 | 2.5 | 4.1 | 4.2 | 4.1 | 0.9 |
| Man6 | 11.0 | 5.0 | 12.8 | 5.3 | 4.1 | 10.4 | 1.9 | 6.6 | 4.1 | 9.1 | 2.4 | 5.8 | 0.0 |
| Man7 | 6.5 | 12.6 | 8.0 | 10.9 | 6.6 | 10.0 | 3.7 | 23.5 | 8.2 | 8.6 | 4.5 | 14.4 | 0.0 |
| Man8 | 16.5 | 12.0 | 18.1 | 15.2 | 7.6 | 20.6 | 7.8 | 18.8 | 9.5 | 21.8 | 10.6 | 16.6 | 0.3 |
| Man9 | 12.0 | 6.5 | 22.0 | 9.0 | 3.2 | 23.0 | 2.6 | 7.6 | 4.1 | 32.3 | 3.8 | 9.2 | 0.2 |
| Man4Gn | 0.0 | 1.5 | 0.0 | 1.5 | 0.0 | 0.0 | 2.1 | 1.0 | 0.0 | 0.0 | 2.4 | 1.2 | 1.2 |
| Man5Gn | 0.0 | 1.1 | 0.0 | 1.6 | 0.0 | 0.0 | 0.8 | 1.1 | 0.0 | 0.0 | 1.1 | 1.2 | 0.0 |

| **CoVA2-15 mAb** | **mIgA1** | | **SIgA1** | | **mIgA2** | | | | **SIgA2** | | | | **IgG** |
|---|---|---|---|---|---|---|---|---|---|---|---|---|---|
| | NLT | NVS | NLT | NVS | NVT | NLT | NIT | NVS | NVT | NLT | NIT | NVS | NST |
| **non glyc.** | 0.5 | 38.5 | 0.9 | 37.8 | 0.0 | 0.3 | 0.0 | 67.6 | 0.0 | 0.7 | 0.0 | 48.6 | 20.5 |
| GnGn | 17.1 | 5.6 | 7.8 | 3.8 | 16.6 | 10.2 | 13.3 | 5.1 | 17.3 | 2.6 | 8.4 | 1.7 | 43.5 |
| MGn | 19.3 | 13.9 | 10.6 | 6.3 | 14.7 | 17.1 | 21.2 | 9.9 | 14.3 | 3.6 | 11.8 | 2.3 | 21.3 |
| MM | 10.2 | 12.1 | 4.5 | 1.9 | 11.2 | 8.0 | 23.4 | 1.7 | 11.0 | 1.7 | 4.0 | 0.5 | 3.1 |
| GnGnF | 0.0 | 4.3 | 0.0 | 1.0 | 3.6 | 0.0 | 2.7 | 3.8 | 3.6 | 0.0 | 0.9 | 0.2 | 0.8 |
| MGnF | 0.0 | 1.4 | 0.0 | 0.5 | 2.5 | 0.0 | 4.7 | 1.2 | 1.9 | 0.0 | 1.3 | 0.3 | 1.3 |
| MMF | 0.0 | 0.0 | 0.0 | 0.0 | 1.5 | 0.0 | 5.2 | 0.0 | 1.0 | 0.0 | 0.4 | 0.0 | 0.0 |
| Man4 | 1.7 | 1.2 | 2.1 | 1.6 | 2.4 | 2.4 | 2.7 | 0.5 | 3.0 | 1.4 | 4.3 | 1.1 | 0.0 |
| Man5 | 1.8 | 2.0 | 2.8 | 3.8 | 3.2 | 3.5 | 3.0 | 1.2 | 5.2 | 2.6 | 6.6 | 2.6 | 3.2 |
| Man6 | 5.4 | 2.0 | 8.7 | 5.1 | 4.8 | 5.3 | 2.0 | 0.9 | 6.1 | 4.9 | 6.2 | 3.8 | 0.0 |
| Man7 | 4.8 | 9.3 | 6.3 | 12.8 | 11.3 | 7.0 | 2.7 | 2.3 | 14.3 | 4.8 | 6.2 | 10.0 | 0.7 |
| Man8 | 23.4 | 6.0 | 23.6 | 15.3 | 21.0 | 21.2 | 11.7 | 2.0 | 16.2 | 19.1 | 25.4 | 15.5 | 1.7 |
| Man9 | 15.8 | 2.4 | 32.7 | 8.5 | 7.4 | 25.0 | 5.5 | 1.9 | 6.1 | 58.5 | 23.1 | 12.5 | 2.1 |
| Man4Gn | 0.0 | 0.8 | 0.0 | 0.8 | 0.0 | 0.0 | 1.5 | 1.2 | 0.0 | 0.0 | 0.8 | 0.4 | 1.9 |
| Man5Gn | 0.0 | 0.4 | 0.0 | 1.0 | 0.0 | 0.0 | 0.4 | 0.8 | 0.0 | 0.0 | 0.6 | 0.5 | 0.0 |

**Table S4: Quantification of the relative abundance [%] of *N*-glycans detected on the secretory component and joining chain of IgA isotypes produced in *N. benthamiana* ΔXT/FT**. *N*-glycans are abbreviated according to the ProGlycAn system (www.proglycan.com). The symbols for the monosaccharides are drawn according to the nomenclature from the Consortium for Functional Glycomics.

| **2E8 mAb** | **SIgA1** | | | | | **SIgA2** | | | | |
|---|---|---|---|---|---|---|---|---|---|---|
| | **SC** | | | | **JC** | **SC** | | | | **JC** |
| | **NDT** | **NYT** | **NGT** | **NVT** | **NIS** | **NDT** | **NYT** | **NGT** | **NVT** | **NIS** |
| **non glycosylated** | 0.0 | 6.3 | 0.0 | 0.0 | 0.0 | 0.0 | 6.2 | 0.0 | 0.0 | 0.0 |
| GnGn | 9.1 | 20.5 | 0.0 | 14.2 | 1.7 | 10.1 | 18.6 | 0.0 | 11.2 | 2.1 |
| GnM | 23.7 | 27.4 | 0.0 | 36.3 | 3.1 | 22.8 | 21.4 | 0.0 | 35.5 | 4.0 |
| MM | 18.9 | 45.8 | 0.0 | 45.9 | 5.4 | 24.1 | 53.9 | 0.0 | 49.8 | 5.7 |
| GnGnF | 0.0 | 0.0 | 0.0 | 0.0 | 0.0 | 0.0 | 0.0 | 0.0 | 0.0 | 0.0 |
| MGnF | 0.0 | 0.0 | 0.0 | 0.0 | 0.0 | 0.0 | 0.0 | 0.0 | 0.0 | 0.0 |
| MMF | 0.0 | 0.0 | 0.0 | 0.0 | 0.0 | 0.0 | 0.0 | 0.0 | 0.0 | 0.0 |
| Man4 | 0.0 | 0.0 | 0.0 | 0.0 | 9.4 | 0.0 | 0.0 | 0.0 | 0.0 | 8.9 |
| Man5 | 0.0 | 0.0 | 3.8 | 0.0 | 11.6 | 0.0 | 0.0 | 4.3 | 0.0 | 16.8 |
| Man6 | 0.0 | 0.0 | 8.5 | 0.0 | 14.9 | 0.0 | 0.0 | 10.2 | 0.0 | 14.5 |
| Man7 | 12.4 | 0.0 | 26.1 | 0.0 | 30.4 | 9.0 | 0.0 | 26.4 | 0.0 | 27.6 |
| Man8 | 26.6 | 0.0 | 41.2 | 0.0 | 15.7 | 21.9 | 0.0 | 38.0 | 9.1 | 13.4 |
| Man9 | 9.3 | 0.0 | 19.9 | 0.0 | 7.7 | 12.0 | 0.0 | 19.1 | 23.7 | 7.0 |
| Man9+Hex | 0.0 | 0.0 | 0.5 | 0.0 | 0.0 | 0.0 | 0.0 | 2.0 | 0.0 | 0.0 |
| MU | 0.0 | 0.0 | 0.0 | 3.5 | 0.0 | 0.0 | 0.0 | 0.0 | 3.4 | 0.0 |

| **CoVA2-15 mAb** | **SIgA1** | | | | | **SIgA2** | | | | |
|---|---|---|---|---|---|---|---|---|---|---|
| | **SC** | | | | **JC** | **SC** | | | | **JC** |
| | **NDT** | **NYT** | **NGT** | **NVT** | **NIS** | **NDT** | **NYT** | **NGT** | **NVT** | **NIS** |
| **non glycosylated** | 0.0 | 11.1 | 0.0 | 0.0 | 0.0 | 0.0 | 19.4 | 0.0 | 0.0 | 0.0 |
| GnGn | 12.9 | 17.4 | 0.0 | 12.7 | 0.9 | 4.9 | 13.4 | 0.0 | 12.1 | 1.1 |
| GnM | 22.3 | 20.2 | 0.0 | 30.9 | 1.6 | 12.8 | 20.6 | 0.0 | 32.2 | 2.0 |
| MM | 27.0 | 51.3 | 0.0 | 53.6 | 4.6 | 19.8 | 46.6 | 0.0 | 53.3 | 4.2 |
| GnGnF | 0.0 | 0.0 | 0.0 | 0.0 | 0.0 | 0.0 | 0.0 | 0.0 | 0.0 | 0.0 |
| MGnF | 0.0 | 0.0 | 0.0 | 0.0 | 0.0 | 0.0 | 0.0 | 0.0 | 0.0 | 0.0 |
| MMF | 0.0 | 0.0 | 0.0 | 0.0 | 0.0 | 0.0 | 0.0 | 0.0 | 0.0 | 0.0 |
| Man4 | 0.0 | 0.0 | 0.0 | 0.0 | 7.4 | 0.0 | 0.0 | 0.0 | 0.0 | 5.8 |
| Man5 | 0.0 | 0.0 | 3.3 | 0.0 | 13.9 | 0.0 | 0.0 | 3.2 | 0.0 | 10.0 |
| Man6 | 0.0 | 0.0 | 7.6 | 0.0 | 11.9 | 0.0 | 0.0 | 7.5 | 0.0 | 10.2 |
| Man7 | 8.7 | 0.0 | 24.4 | 0.0 | 22.1 | 11.9 | 0.0 | 21.7 | 0.0 | 20.2 |
| Man8 | 22.4 | 0.0 | 37.8 | 0.0 | 17.2 | 36.4 | 0.0 | 33.2 | 12.9 | 20.8 |
| Man9 | 6.7 | 0.0 | 26.7 | 0.0 | 20.4 | 14.2 | 0.0 | 32.8 | 22.3 | 25.9 |
| Man9+Hex | 0.0 | 0.0 | 0.3 | 0.0 | 0.0 | 0.0 | 0.0 | 1.5 | 0.0 | 0.0 |
| MU | 0.0 | 0.0 | 0.0 | 2.8 | 0.0 | 0.0 | 0.0 | 0.0 | 2.5 | 0.0 |

**Table S5: Modifications of the hinge-region of plant-produced COVA2-15 and 2E8 monomeric and secretory IgA1.** Relative quantification (%) of hydroxyprolines and arabinose chains in the hinge-region tryptic peptide.

| | 2E8 mIgA1 | 2E8 SIgA1 | COVA2-15 mIgA1 | COVA2-15 SIgA1 |
|---|---|---|---|---|
| 0 hyP + no Ara | 7.3 | 8.2 | 2.4 | 3.5 |
| 1 hyP + no Ara | 9.5 | 11.6 | 7.1 | 10.4 |
| 2 hyP + no Ara | 12.6 | 14.5 | 15.2 | 21.4 |
| 3 hyP + no Ara | 11.7 | 11.6 | 16.6 | 21.4 |
| 4 hyP + no Ara | 5.3 | 4.8 | 11.3 | 13.6 |
| 5 hyP + no Ara | 1.5 | 1.4 | 5.8 | 6.0 |
| 4 hyP + 1 Ara | 2.0 | 1.6 | 1.5 | 1.1 |
| 5 hyP + 1 Ara | 2.3 | 2.5 | 2.1 | 1.4 |
| 6 hyP + 1 Ara | 0.8 | 0.7 | 0.3 | 0.3 |
| 4 hyP + 2 Ara | 2.3 | 2.2 | 1.8 | 1.2 |
| 5 hyP + 2 Ara | 1.5 | 2.6 | 2.7 | 1.8 |
| 6 hyP + 2 Ara | 0.4 | 0.9 | 1.0 | 0.2 |
| 4 hyP + 3 Ara | 3.1 | 3.8 | 1.8 | 1.1 |
| 5 hyP + 3 Ara | 3.0 | 3.6 | 3.7 | 2.0 |
| 6 hyP + 3 Ara | 2.7 | 1.8 | 1.6 | 1.1 |
| 4 hyP + 4 Ara | 1.6 | 2.3 | 1.8 | 1.1 |
| 5 hyP + 4 Ara | 3.0 | 3.0 | 3.7 | 1.6 |
| 6 hyP + 4 Ara | 2.1 | 1.8 | 1.0 | 0.8 |
| 4 hyP + 5 Ara | 2.1 | 1.9 | 1.0 | 0.8 |
| 5 hyP + 5 Ara | 3.8 | 2.9 | 2.5 | 1.4 |
| 6 hyP + 5 Ara | 3.1 | 2.0 | 2.2 | 0.9 |
| 4 hyP + 6 Ara | 3.6 | 2.9 | 2.0 | 1.0 |
| 5 hyP + 6 Ara | 3.2 | 2.6 | 2.7 | 1.7 |
| 6 hyP + 6 Ara | 1.9 | 2.0 | 1.7 | 0.8 |
| 4 hyP + 7 Ara | 1.8 | 1.5 | 1.0 | 0.5 |
| 5 hyP + 7 Ara | 2.2 | 1.2 | 1.6 | 0.9 |
| 6 hyP + 7 Ara | 1.9 | 1.7 | 1.7 | 0.9 |
| 4 hyP + 8 Ara | 1.3 | 0.6 | 0.3 | 0.2 |
| 5 hyP + 8 Ara | 1.4 | 1.0 | 1.0 | 0.4 |
| 6 hyP + 8 Ara | 1.0 | 0.8 | 0.9 | 0.5 |

**Table S6: $EC_{50}$ values of COVA2-15 and 2E8 antibody variants for binding to SARS-CoV-2 VLPs determined by ELISA**. Each value is the mean ± standard deviation from at least two independent measurements.

| | $EC_{50}$ [nM] | | | | |
|---|---|---|---|---|---|
| | IgG | mIgA1 | mIgA2 | SIgA1 | SIgA2 |
| | COVA2-15 | | | | |
| **Wuhan** | 0.032 ± 0.011 | 0.054 ± 0.030 | 0.149 ± 0.050 | 0.010 ± 0.018 | 0.017 ± 0.009 |
| **Delta** | 9.028 ± 0.019 | 1.951 ± 0.014 | 2.063 ± 0.068 | 0.086 ± 0.026 | 0.542 ± 0.036 |
| | 2E8 | | | | |
| **Wuhan** | 0.271 ± 0.143 | 0.216 ± 0.030 | 0.836 ± 0.023 | 0.002 ± 0.123 | 0.005 ± 0.068 |
| **Delta** | 1.071 ± 0.133 | 0.439 ± 0.029 | 2.031 ± 0.029 | 0.023 ± 0.030 | 0.029 ± 0.028 |

**Table S7:** Kinetic parameters of COVA2-15 and 2E8 monomeric IgA mAbs to RBD. Rate constants were determined at 5 different concentrations using a 1:1 binding model.

| | ka (1/Ms) | kd (1/s) | *KD* (nM) |
|---|---|---|---|
| **COVA2-15 mIgA1** | 230920.6 | 0.00027 | 1.43 |
| **COVA2-15 mIgA2** | 207058.8 | 0.00035 | 1.67 |
| **2E8 mIgA1** | 79590.0 | 0.00100 | 125.42 |
| **2E8 mIgA2** | 97574.8 | 0.00085 | 89.59 |

**Table S8: $ID_{50}$ values of COVA2-15 and 2E8 antibody variants for neutralization of SARS-CoV-2 (England 02/2020).** Neutralisation capacity was measured using a PRNT assay on Vero E6 cells. mAbs were added in serial 1:10 dilutions starting with 10 µg/mL. A positive control (Pos; WHO International Standard of anti-SARS-CoV-2 immunoglobulin, 20/136, NIBSC, UK) was included. $IC_{50}$ vales have been determined from duplicates of one representative out of two experiments with similar results using non-linear regression in GraphPad Prism.

| | IC50 [ng/mL] | | |
|---|---|---|---|
| **mAb** | **value** | **+error** | **-error** |
| Positive control | 76.1 | 24,0 | 18.2 |
| CoVA2-15 IgG | 53.4 | 6.9 | 6.1 |
| CoVA2-15 mIgA1 | 29.7 | 6.9 | 5.6 |
| CoVA2-15 SIgA1 | 13.5 | 3.6 | 2.8 |
| CoVA2-15 mIgA2m(1) | 81.2 | 8.2 | 7.5 |
| CoVA2-15 SIgA2m(1) | 22.8 | 5.5 | 4.4 |
| 2E8 IgG | | n.d | |
| 2E8 mIgA1 | 1707.0 | 902.8 | 590.6 |
| 2E8 SIgA1 | 36.6 | 37.4 | 18.5 |
| 2E8 mIgA2m(1) | 3210.1 | 4482.5 | 1870.5 |
| 2E8 SIgA2m(1) | 176.1 | 149.4 | 80.8 |
